# Size-exclusion chromatography as a clinically applicable method for extracellular vesicles collection as liquid biopsies shows potential diagnostic relevance and correlation with poorer outcome in common cancer types

**DOI:** 10.1016/j.tranon.2026.102964

**Published:** 2026-08-04

**Authors:** Szilárd-Krisztián Belényesi, Seán Patmore, Sinead Hurley, Ezgi Oner, Volga M. Saini, Marika Kanjuga, Faye Lewis, Meghana S. Menon, Christina Cahill, Antonia Tierney, Oana Deac, Maria Jose Santos Martinez, Maeve A. Lowery, John J. O’Leary, Kathy Gately, Sharon O’Toole, Lorraine O’Driscoll

**Affiliations:** a School of Pharmacy and Pharmaceutical Sciences, Trinity College Dublin, Dublin, Ireland; b Trinity Biomedical Sciences Institute, Trinity College Dublin, Dublin, Ireland; c Trinity St. James’s Cancer Institute, Trinity College Dublin, Dublin, Ireland; d Department of Obstetrics and Gynaecology, School of Medicine, Trinity College Dublin, Ireland; e Department of Histopathology, School of Medicine, Trinity College Dublin, Dublin, Ireland; f Trinity Translational Medicine Institute, St James’s Hospital, Dublin, Ireland; g Department of Clinical Medicine, School of Medicine, Trinity College Dublin, Ireland; h Department of Oncology, St James's Hospital, Dublin, Ireland

**Keywords:** Liquid biopsy, Circulating biomarkers, Extracellular vesicles, Diagnostic, Cancers: ovarian, Lung, Gastric, Patients’ outcome

## Abstract

Increasing evidence suggests extracellular vesicles (EVs) are key components of liquid biopsies to aid cancer diagnosis and treatment selection, if their collection can be translated to clinical settings. Size exclusion chromatography (SEC) is relatively simple, fast, and cost-effective, but its potential for “real-world” EVs collection for cancer diagnoses is unknown. Here we aimed to critically evaluate SEC using clinically-relevant plasma volumes and across a range of common cancer types that are frequently diagnosed too late. EVs were collected, using qEV70 or qEV35 SEC columns, from duplicate aliquots of n = 43 plasma samples procured at diagnosis with lung, ovarian, or gastric cancers and characterised on multiple parameters, seeking correlations with patients clinicopathological information. To investigate potential diagnostic relevance, EVs were collected in the same way from n = 10 plasma samples from healthy donors. We found that SEC has many attributes indicating that it is translatable to real-world clinical utility. With both SEC types, relatively large quantities of EVs were successfully collected from small plasma volumes for all three cancer types. No significant differences existed between qEV70 and qEV35 particle sizes, structure, and EVs markers. EVs in qEV35 isolates versus qEV70 isolates had reduced purity and/or increased protein corona, but both isolates revealed clinical correlations. Importantly, depending on the cancer type(s), results suggested that the EVs isolates at the time of cancer diagnosis may inform on lymph node-positivity and advanced stage, and may be prognostic for shorter time to disease progression. Furthermore, analysis of plasma CD63+ EVs and CD81+ EVs appears to have diagnostic relevance, due to their typically significantly greater quantities in cancer types compared to healthy controls. Altogether, SEC shows promise as a potential approach for clinical EVs collection as liquid biopsies and correlations between plasma EVs quantities with a range of clinicopathological characteristics suggests this would be worthwhile.

## Highlights

• Size-exclusion chromatography pulls extracellular vesicles from “real world” plasma.
• Different chromatography columns offer some complementary diagnostic and prognostic information.
• Notwithstanding some methodology limitations, this is applicable in many cancers.
• This method can bridge laboratory research to the clinical setting.

## Introduction

Blood tests, instead of invasive tissue biopsies, are the holy grail for cancer diagnosis and optimal treatment selection for patients. Such blood tests could be faster, cheaper, and routinely available, avoiding unnecessary delays for those with cancer and worry for those with harmless conditions. These blood tests may also assist with optimal treatment choices, while avoiding use of often expensive drugs producing side-effects but no benefit; and reducing overall strain on clinics/healthcare. And even where liquid biopsies could not replace tissue biopsies, undoubtedly they could add additional benefit by enabling on-going monitoring for tumour recurrence, treatment response, etc., contributing to improved quality-of-life and longevity. In 2010, the term “liquid biopsy” was initially coined to describe the circulating tumour cells components of blood tests [1]. However, we know from our previous research and that of others [[2], [3], [4]] that extracellular vesicles (EVs) are also a potentially very valuable component of liquid biopsies.

Extracellular vesicles (EVs) are small lipid bilayer-surrounded particles released by most, if not all, cells in the body and primarily found within bodily fluids such as blood [5,6]. EVs have been described as ‘mini-maps’ of their cells of origin and are involved in cell-to-cell communication [7]. As indicated above, there is growing optimism from our research and that of others that the assessment of EVs from biofluids may represent a relatively quick and minimally-invasive “liquid biopsy” [[8], [9], [10], [11]]. And of the potential biofluids that could act as a liquid biopsy source, blood constitutes a very promising option for evaluating cancer patients’ EVs, given that blood samples are routinely procured from patients for on-going monitoring e.g. of white blood cell counts. Thus, in standard-of-care practice, assigning a blood sample for EVs analysis would neither add stress for the patient nor extra work for the nurse/phlebotomist drawing the blood. Furthermore, EVs (of 30–1000 nm size range) are reported to exist in the blood plasma at concentrations of up to 10^10^ EVs/mL [12], giving confidence that relativity small volumes of blood plasma would be adequate for routine EVs testing in the future, when methods for EVs collection can be translated to clinical utility.

A range of methods are options for collecting EVs fractions from biofluids. These methods include ultracentrifugation, tangential flow filtration, immunomagnetic beads, and size exclusion chromatography (SEC) [13]. However, challenges are met when aiming to bridge laboratory research on EVs to the clinical setting when methods require complex and expensive equipment; are time consuming; and/or involve multiple steps, especially as each typically reduces EVs yield, making analysis from small sample volumes impractical. Thus, with a specific focus on future clinical utility rather than fundamental research, our study focused on methods that require small volumes – as cancer patients’ samples are precious and often limited in volume – and require minimal equipment, complexity, resources and time. Considering the views of practicing clinicians routinely performing blood tests as standard of care, immunomagnetic beads and SEC were shortlisted. However, given the bias of immunomagnetic beads to specific EVs’ sub-populations with known surface antigens, SEC was deemed the method that best meets the practical clinical needs.

To remove concerns of SECs not being standardised when poured in-house, 2 types of ISO certified commercially available columns were used i.e. qEV1 70 nm (qEV70) and qEV1 35 nm (qEV35) columns. The latter are described by the manufacturer, IZON (izon.com) as having a smaller resin pore size than the former and so optimal for higher recovery of 35–350 nm size range (i.e. “smaller EVs”) compared to those of 70–1000 nm size range (i.e. “larger EVs”) for the qEV70, but also with the caveat of more so-called contaminating proteins in qEV35 isolates. Here we report the first study of its kind characterising and comparing qEV70 and qEV35 isolates from a broad range of “real world” plasma samples from patients at the time of diagnosis with three cancer types that are increasingly common and that frequently result in poor outcomes for patients (i.e. lung, ovarian, and gastric cancers). While some other researchers compared SEC columns [[14], [15], [16], [17], [18]], those studies used plasma from either healthy controls [14,15], or a single cancer type [16] and, crucially, often using pooled plasma rather than individual patient samples [17,18], which allow important fundamental research but would not be appropriate in a clinical setting. Our study uniquely considered differences between individual plasma samples, collected from a heterogeneous cohort of patients with common cancers, pre-processed using biobanking standard operating procedures, and in a routine clinical setting.

## Patients and methods

### Patient recruitment and sample collection

Written informed consent was obtained from all patients prior to participation, and the studies were approved by the St James’s Hospital (SJH)/Tallaght University Hospital Joint Research Ethics Committee and in compliance with the General Data Protection Regulation (GDPR) and Data Protection Act 2018. Blood samples (9 mL) were procured from 43 treatment-naïve patients, with lung (n = 17), ovarian (n = 17), or gastric (n = 9) cancer. The lung cancer patients included 9:8 (53:47%) females:males, with median age 71y; the gastric cancer patients included 3:6 (33.3:66.6%) females:males, with median age 64y; while the 17 females with ovarian cancer had a median age of 56y.

In keeping with the accepted standard operating procedures available at the time of blood collection and to keep the study as “real world” as possible, blood from patients diagnosed with lung cancer or ovarian cancer was collected into K2-EDTA 9 mL tubes (Greiner Bio-One, Austria, Cat. #455,045), while for gastric cancer, ACD-A 9 mL tubes were used (Greiner Bio-One, Austria, Cat. #455,055). As platelets are a very rich source of EVs, procedures were applied to eliminate/minimise platelets presence i.e. to produce platelet-depleted plasma (PDP). Specifically, once blood was procured, it was centrifuged at 2500 x g for 15′ at room temperature (RT), with no brake. The supernatant was then collected ∼100 μL above the pellet and placed in a new tube which was centrifuged at 2500 x g for 15′ at RT, no brake. For lung and ovarian cancer patients, the plasma was filtered after the second centrifugation using 0.8 μm filter paper (Fisherbrand, Fisher Scientific, Ireland, Cat. #10,556,722), while gastric cancer samples were not given this extra filtration step. The resulting PDP was aliquoted into biobanking tubes (Sarstedt, Germany, Cat. #72.694.006) and stored at −80 °C for analysis.

To investigate potential diagnostic relevance of the EVs, written informed consent was obtained from n = 10 healthy donors (i.e. donors with no history of cancer or known presence of cancer), following the study’s approval by the School of Pharmacy and Pharmaceutical Sciences Ethics Committee, and in compliance with the GDPR and Data Protection Act 2018. The cohort included 5:5 (50%:50%) females: males. Blood samples were collected into K2-EDTA 9 mL tubes (Greiner Bio-One, Austria, Cat. #455,045), as for the lung cancer and ovarian cancer samples. The blood was centrifuged at 2500 x g for 15′ at room temperature (RT), with no brake. The supernatant was then collected ∼100 μL above the pellet and placed in a new tube which was centrifuged at 2500 x g for 15′ at RT, no brake. The plasma was then filtered using 0.8 μm filter paper (Fisherbrand, Fisher Scientific, Ireland, Cat. #10,556,722), aliquoted into biobanking tubes, and stored at −80 °C for analysis.

### Collection of extracellular vesicles using size exclusion chromatography

**Fig. 1:**
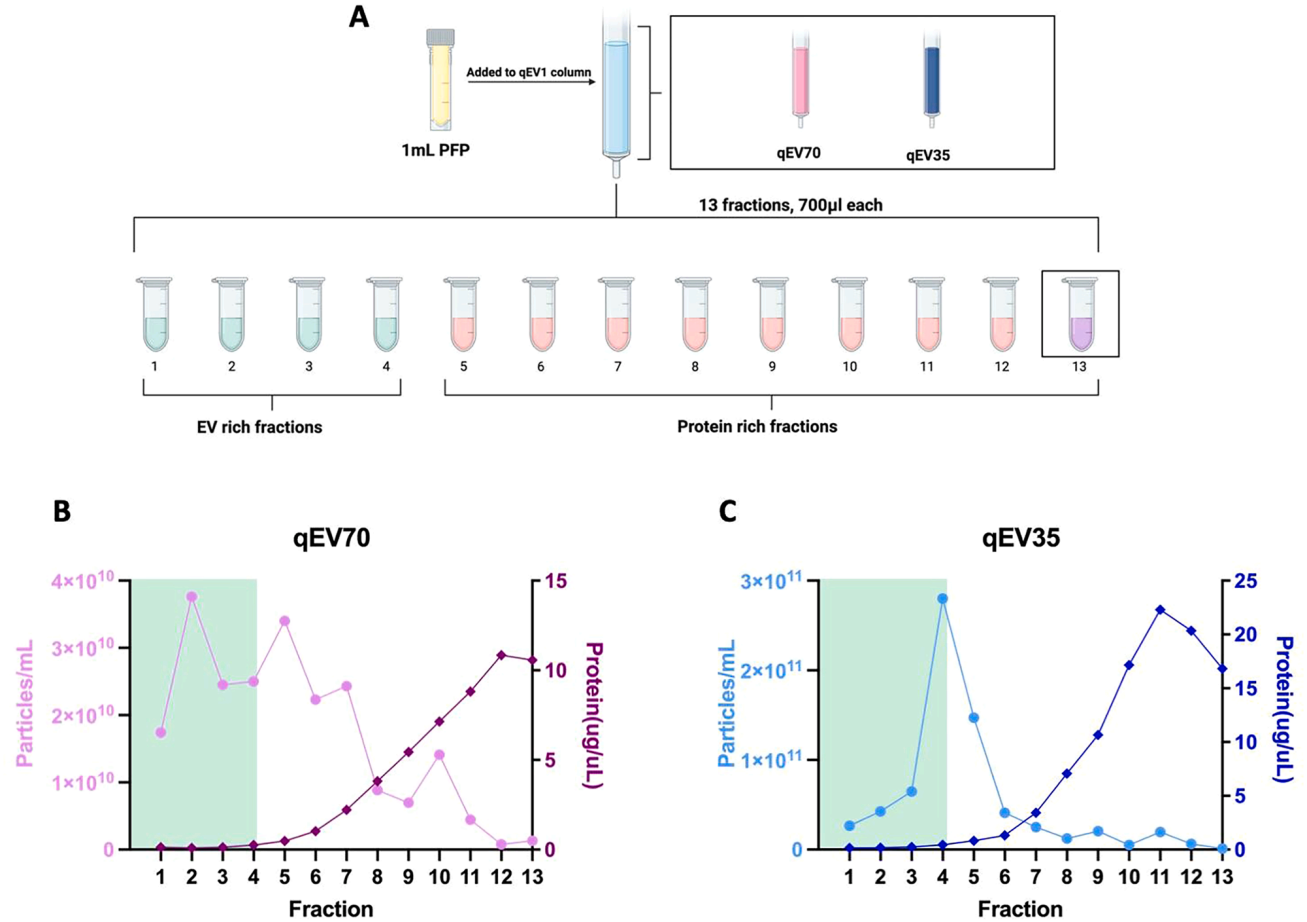
**Collection and characterisation of fractions from qEV70 and qEV35 SEC.** Platelet-depleted plasma (PDP) was loaded onto either qEV70 or qEV35 columns, and 13 fractions collected (A). NTA determine particles concentrations was graphed with Bradford assay determined protein concentrations, for both qEV70 (B) and qEV35 (C) isolates.

Size-exclusion chromatography (SEC) columns qEV1 35 nm (qEV35, IZON, France, Cat. # IC1–35) or qEV1 70 nm (qEV70, IZON, France, Cat. #IC1–70) were calibrated and flushed, according to manufacturer’s instruction. For each patient, identical pairs of plasma aliquots (1 mL each) were loaded onto a qEV35 or qEV70 column, respectively, and then each topped up with 12.5 mL double-filtered PBS. A consistent buffer volume (BV) of 4.7 mL was discarded, and 13 total fractions of 700 μL each were collected using an automated fraction collector (Fig. 1. A). Based on the manufacturer’s instructions and validated experimentally (Fig. 1. B, C), the first four fractions (i.e. the EVs-rich fractions) were pooled for downstream analysis. Incidentally, the 13th fraction (F13, containing extravesicular proteins, but no EVs) was kept as a protein control. Pooled isolates of fractions 1–4 were spin-concentrated using the Amicon 30 kDa MWCO filter tubes (Merck Millipore, Germany, Cat. #UFC803024), centrifuging at 3260 x g at 4 °C, in increments to a final volume of ∼250 μL. To avoid freeze-thaw cycles of the EVs isolates, the pooled fractions were aliquoted and stored at −80 °C for subsequent characterisation.

### Transmission electron microscopy

For transmission electron microscopy, as we previously described [19], EVs isolates (10 μL) were placed onto carbon-coated grids (Ted-Pella B 300 M, Mason Technology Ltd., Cat. #01,813-F) and incubated at RT for 10 min. After incubation, the samples were fixed using 4% glutaraldehyde and contrasted with 2% phospho-tungstic acid. The grids were examined at 100 kV using a JEOL JEM-2100 TEM (JEOL USA Inc.).

### Nanoparticle tracking analysis

EVs isolates (50 μL) were diluted with double-filtered PBS, as required to achieve an optimal dilution, with optimal deemed to be when the NTA reported “optimal capture conditions”. The samples were loaded into the NTA system using a NanoSight syringe pump. A NanoSight Pro system (Malvern Instruments Ltd.) was used to determine particle concentrations and average size distribution of the isolates. Brownian motion of the particles was captured at a laser wavelength of 488 nm, at a flow rate of 3μL/min. Five consecutive captures were taken and analysed using the NanoSight Pro 1.0.0.6 software. Aliquots of the filtered PBS were used as negative control.

### Lysis of EVs aliquots for immunoblotting and ELISAs

EVs isolates (100 μL) had an equal volume of lysis buffer (Invitrogen, Massachusetts, United States, Cat. #FNN0011) added, along with 1:100 protease inhibitor Halt™ Protease Inhibitor Single-Use Cocktail 100X (ThermoFisher, Ireland; Cat. #78,430). Lysed samples were centrifuged at 16,100 x g for 10 min at 4 °C. Protein was quantified using Bio-Rad Bradford’s Reagent (BioRad Laboratories, California, US; Cat. #5000,006).

### Immunoblotting

For immunoblotting, EVs isolates (20 μg) or whole cell lysates (10 μg WCL) were loaded onto 4–20% gradient Mini-PROTEAN® TGXTM Precast Protein Gels (BioRad Laboratories, California, United States, Cat. #4561,094) for electrophoresis. Each gel was run with samples prepared under two conditions: reduced (using beta-mercaptoethanol as reducing agent), and non-reduced (where beta-mercaptoethanol was substituted with PBS). Molecular weight marker Precision Plus Protein™ Kaleidoscope™ Pre-stained Protein Standards (BioRad Laboratories, California, United States, Cat. #1610,375) were run in parallel with samples. Separated proteins were transferred onto an immuno-blot PVDF membrane (BioRad Laboratories, California, United States, Cat. #1620,174).

Membranes were incubated with primary antibodies: anti-syntenin (1:500, Abcam, Netherlands, Cat. #ab19903), anti-CD9 (1:500, Abcam, Netherlands, Cat. #ab236630), anti-calnexin (1:1000, Abcam, Netherlands, Cat. # ab133615), anti-GRP94 (1:1000, Cell Signalling, Netherlands, Cat. #2104S), or anti-albumin (1:2000, Abcam, Netherlands, Cat. #ab207327). HRP-linked anti-rabbit IgG was used as a secondary antibody for all samples (Cell Signalling, Cat. #7074S). The membranes were imaged using a ChemiDoc exposure system (BioRad Laboratories, California, United States) where SuperSignal® West Pico Chemiluminescence Substrate (ThermoFisher, Ireland, Cat. #34,080) or SuperSignal® West Femto Maximum Sensitivity Substrate (ThermoFisher, Ireland, Cat. #3409 s) were used. ImageJ software was used for densitometry.

### Apolipoprotein B ELISA

Apo B in EVs isolates (250ng/well) was quantified using an ApoB ELISA kit (Abcam, Netherlands, Cat. # ab190806), following manufacturer’s instructions. WCL was used as a control.

### Flow cytometric analysis of EVs

Paired EVs samples were evaluated by flow cytometry using a calibrated Cytek® Aurora full-spectrum flow cytometer (Cytek Biosciences, Fremont, CA, USA). The flow cytometer was calibrated for EVs detection using gaintration, swarming detection, NIST size standards 100 nm (Thermo Fisher Scientific, USA, Cat. #3100A) and 150 nm (Thermo Fisher Scientific, USA, Cat. #3150A), with a template for analysis established following these calibration steps. Gaintration tests were used to identify peak separation between background noise and signal. Swarming was controlled for by running dilution experiments to identify the point of linearity in each sample type tested (SEC fractions, qEV35 and 70 nm). Antibody concentrations were calculated by performing a series of dilutions, CD9 (1:80, ExBio, Czechia, Cat. #1P-208-T100, Clone MEM-61, PE), CD63 (1:80, Beckman Colter, USA, Cat. #IM1914U, PE), CD81 (1:80, ExBio, Czechia, Cat. #1P-558-T025, Clone M38, PE), as well as 1000X CellMaskTM Green Plasma Membrane Stain (1:1100, Invitrogen, Massachusetts, United States, Cat. #: C377608). Prior to each run, the flow cytometer was calibrated using Rosetta calibration beads (Exometry, Netherlands, Cat. #CAL003) and polystyrene size standard 100 nm and 150 nm. Flow rate was set on the lowest speed, and all samples ran for 2 min. The flow rate was calculated using Apogee beads (Apogee, UK, Cat. #1493). Total EVs concentration per sample was calculated by the formula (count x dilution factor x flow rate). Of note: an in-depth explanation of the flow cytometry methodology and gating strategy for EVs analysis can be found in the Supplementary Material: Supplemental Method.

### Statistical analysis

All statistical analysis was performed using GraphPad Prism 10.5.0 (GraphPad Software, Dotmatics, Boston, MA, USA), with further detail in figure legends. P values <0.05 were considered statistically significant. The normality of each data set was assessed with a Shapiro-Wilk test. Where both data sets involved in an analysis met the assumption of normality, a parametric test (paired T-test) was performed. Where data was non-normally distributed, a non-parametric test was performed (Wilcoxon and Kruskal-Wallis tests for pairwise comparisons, Spearman’s test for correlations with Holm-Šidák correction applied as relevant consider relatively small sample size). Mann-Whitney tests were applied to compare EVs abundance in plasma from cancer patients versus healthy donors. Survival analysis was performed using Kaplan-Meier survival curves and Cox regression. In the case of Spearman’s test, a r_s_>0 was considered a positive association, and an r_s_<0 was considered negative. The magnitude of Spearman’s correlation was considered as follows: 0.00–0.19, very weak; 0.20–0.39, weak; 0.40–0.59, moderate; 0.60–0.79, strong; and 0.80–1.00, very strong.

## Results

### Determining EVs-rich fractions and collecting for all plasma samples

Prior to advancing to collect EVs from all the plasma samples, NTA and protein quantification were used to determine which SEC fractions were rich in EVs and proteins, by analysing duplicate aliquots of representative plasma samples processed through the qEV70 or the qEV35 columns, respectively. After discarding the void buffer volume, the first four consecutive fractions were found to contain the highest concentrations of particles and lowest concentrations of protein and, thus, were considered the EVs-rich fractions (Fig. 1. B, C) and so collected as EVs isolates for all n = 43 plasma samples and n=10 healthy donors samples. Fractions 5–13 were determined to be particles-poor and protein-rich.

### Characterisation of EVs

**Fig. 2:**
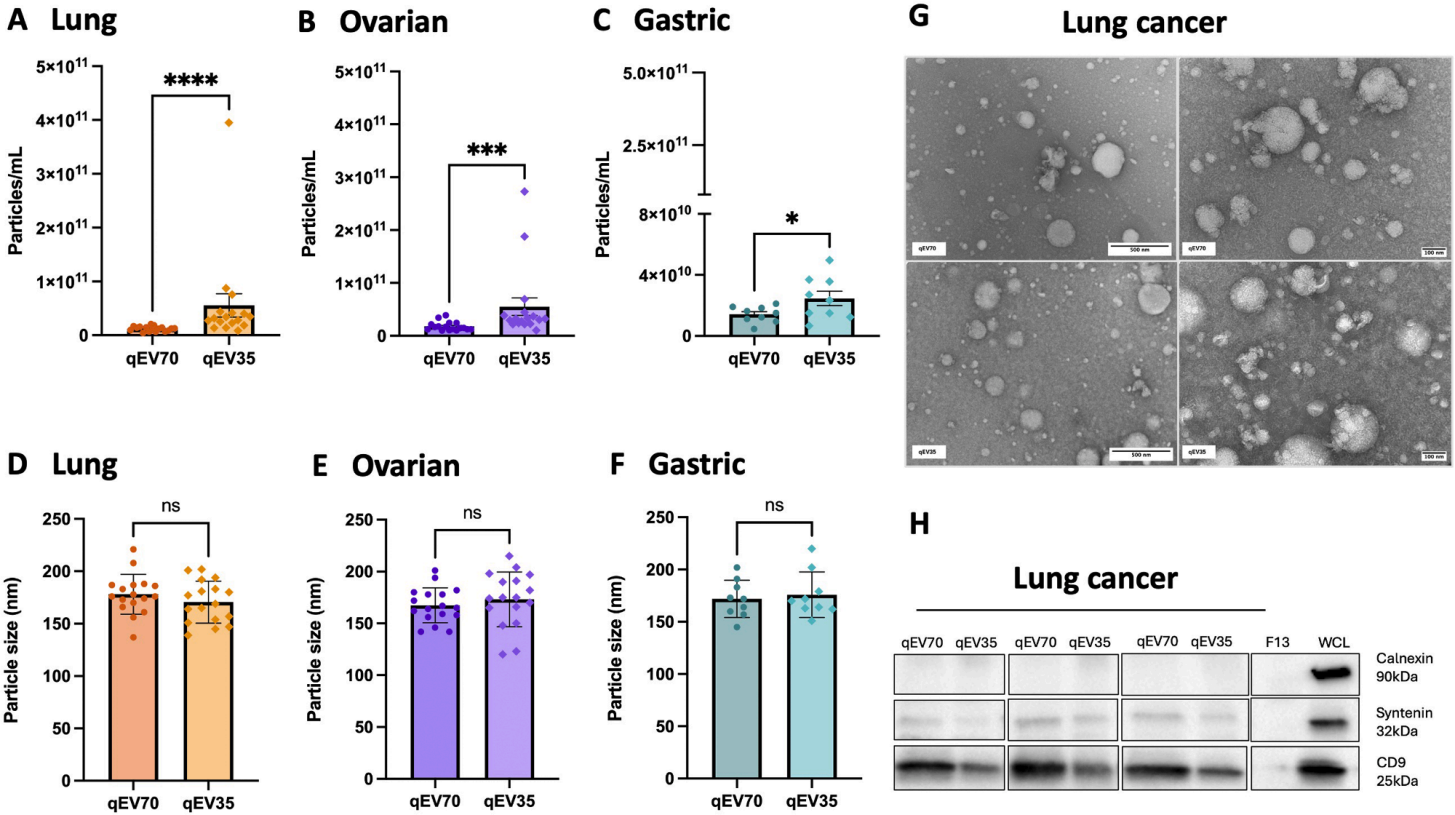
**Characterisation of EVs (qEV70 and qEV35) isolates from the three cancer patient cohorts**. Particles concentrations in the isolates from qEV35 and 70 nm columns were determined by NTA for each cancer type: (A) lung, (B) ovarian, and (C) gastric cancer patients’ plasma EVs (Mean ± SEM, Wilcoxon test). Particles sizes were also determined for each cancer type (D) lung, (E) ovarian, and (F) gastric cancer (Mean ± SD, paired *t*-test). (G) TEM images of EVs, showing gastric cancer as example, with 500 nm and 100 nm scale bars displayed (see Supplementary Fig. 1(A) & (B) for representative images of the lung and ovarian cancer EVs isolates). (H) Immunoblotting detected EVs positive markers (CD9, Syntenin) and did not detect EVs negative markers (Calnexin or GRP94). WCL (whole cell lysate) was used as a control. While the immunoblots are representative of for n = 3 lung cancer patients’ EVs, this analysis was performed for all cancer patients (see Supplementary Fig. 1(C) & (D) for representative immunoblots of the ovarian and gastric cancers EVs isolates). Statistical significance is indicated as *p<0.05, ***p<0.001, ****p<0.0001.

NTA determined particle concentrations and sizes of EVs isolates for all plasma samples, following collection via both qEV columns. In all cases, significant differences were found between the quantities of EVs isolates resulting from using the qEV70 versus qEV35 columns (Fig. 2. A-C). Specifically, the qEV35 columns, compared to the qEV70 columns, resulted in significantly more particles collected per mL of plasma. This was observed for all of the three cancer types i.e. lung cancer 3.41 × 10^10^ vs 1.17 × 10^10^ particles/mL (p < 0.0001); ovarian cancer 2.91 × 10^10^ vs 1.87 × 10^10^ particles/mL (p = 0.0009); and gastric cancer 2.46 × 10^10^ vs 1.42 × 10^10^ particles/mL (p = 0.0234). Interestingly in relation to particles size, no significant differences in resulting EVs isolates existed regardless of the SEC type used. This was consistent for all cancer types. For lung cancer, average particle sizes were 177.4 ± 19.5 nm for qEV70 and 172.1 ± 19.74 nm for qEV35 (Fig. 2D). Similar size distributions were observed in ovarian cancer (Fig. 2E) and gastric cancer (Fig. 2F).

Transmission electron microscopy showed the presence of round-shaped structures representing vesicles in all patients’ sample following EVs collection via qEV70 or qEV35 EVs columns (lung cancer TEM results are exemplified in Fig. 2G; those for ovarian and gastric cancers are represented in Supplemental Fig. 1A & B). In keeping with this, immunoblots detected established EVs positive markers (CD9 and syntenin), while the negative EVs markers calnexin or GRP94 were undetected in isolates from all patients’ samples, regardless of the type of column used (lung cancer immunoblots are exemplified in Fig. 2H; those for ovarian cancer and gastric cancer can be found in Supplemental Fig. 1C & 1D). Some patient-to-patient variability was observed on analysis of EVs markers CD9 and syntenin, but it must be considered that immunoblot is only a semi-quantitative method and is used here rather to support the presence of EVs and in keeping with MISEV2023 guidelines [13], rather than making substantial claims about relative amounts of proteins.

### Assessment EVs isolates contaminants and purity

**Fig. 3:**
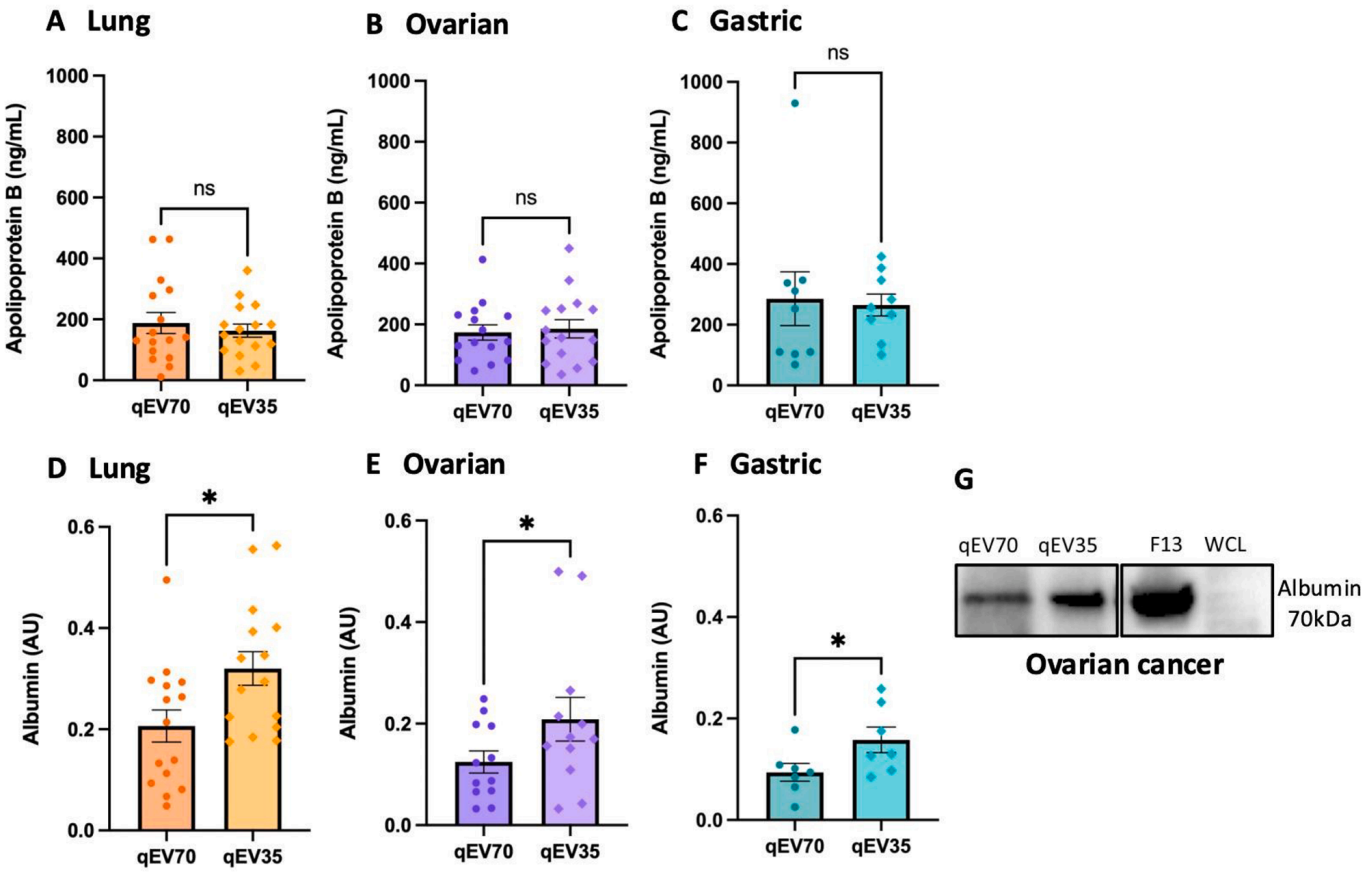
**Assessment of lipoprotein and albumin with EVs collected from three cancer patient cohorts, using two different SEC (qEV70 and qEV35) columns***.* ELISAs detected apolipoprotein B in isolates from plasma of patients with (A) lung, (B) ovarian, and (C) gastric cancer, but with no significant differences detected whether the EVs isolates were collected using qEV70 or qEV35*.* Similarly, immunoblotting detected albumin in EVs isolates for each of (D) lung, (E) ovarian, and (F) gastric cancer. For all three cancer types, statistical analysis of the associated densitometry analysis indicated significantly more albumin was present when qEV35 were used compared to qEV70. (G) Representative immunoblot showing albumin in ovarian cancer EVs isolates, with fraction 13 and whole cell lysate (WCL) controls (see Supplementary 2. B, C, D for all three cancer types). Wilcoxon test showed significance *p<0.05.

So called “contaminants” [20] can potentially collect with EVs (i.e. non-EVs associated proteins and lipoprotein) using SEC, due to their size similarities with EVs. Thus, we assessed EVs collected with both qEV35 and qEV70 for the presence of commonly described plasma contaminants, Apo B and albumin. qEV35 and qEV70 isolates, analysed by ELISA, showed no significant differences in the amount of Apo B that co-isolated with EVs, regardless of samples being from patients with lung, ovarian, or gastric cancer (Fig. 3. A-C). Indeed, when data from each cancer type was combined, we found that EVs isolates from each cancer exhibited similar base levels of Apo B contamination, with no significant difference existing where qEV35 and qEV70 columns were used (Supplemental Fig. 2). Regarding albumin, EVs isolates from plasma of patients with lung, ovarian and gastric cancer showed significantly more albumin when collected with qEV35 compared to qEV70 columns (Fig. 3D-G). This supports the assumption that EVs collected using qEV35 columns have more protein contamination compared to those from qEV70 columns. However, it seems that they do not have significantly more lipoprotein.

Webber and Clayton [21] described a method for estimating an EVs purity ratio based on quantity of particles/µg of protein i.e. particles/mL determined by NTA and dividing by the total amount of protein. Here we found, across all cancer types, that the protein quantities were significantly greater in the qEV35 EVs isolates versus qEV70 EVs isolates i.e. for lung cancer: 418 vs 87.17µg/mL (p < 0.0001); for ovarian cancer: 366.75 vs 92.9µg/mL (p < 0.0001)*,* and for gastric cancer: 252 vs 86.49µg/mL (p = 0.0001) (Supplementary Fig. 3. A-C). Taking the EVs quantities by NTA into account, only for lung cancer were qEV35 isolates found to be significantly (p = 0.0129) less pure than those from qEV70 columns (qEV70 purity estimate was 2.0E+08 ± 1.6E+08; qEV35 purity estimate was 1.2E+08 ± 1.6E+08; denoting a relative 38% decrease in the purity of qEV35-derived EVs isolates). For ovarian and gastric cancers, reductions in purity were found with qEV35, but these were not statistically significant (ovarian: 30% reduction, p = 0.1901; gastric cancer: 46% reduction, p = 0.0977) (Supplementary Fig. 3. D-F).

### Flow cytometric analysis of EVs quantities by assessing EVs markers

**Fig. 4:**
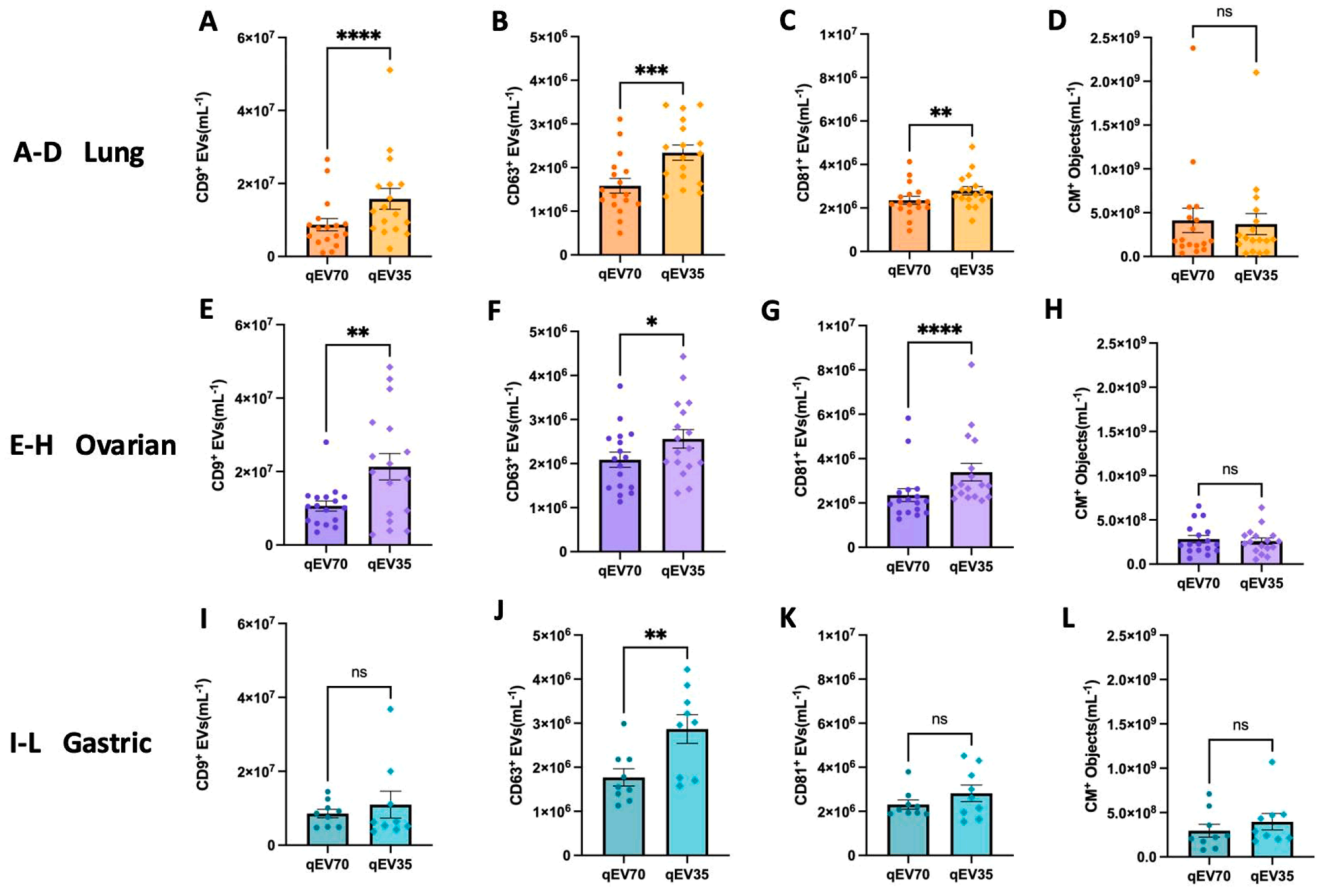
**Flow cytometry analysis of EVs surface markers.** EVs isolates from the qEV70 and qEV35 were evaluated for specific EVs associated tetraspanins and with cell mask (CM) lipid membrane stain, by flow cytometry. Again, comparing identical EVs isolates of the samples but collected using either the qEV70 or qEV35 column type, CD9+ EVs, CD63+ EVs, CD81+ EVs, and cell mark (CM)+ EVs, respectively, were quantified in the (A-D) lung, (E-H) ovarian, and (I-L) gastric patients’ samples. qEV35 columns always resulted in more EVs being collected, considering the EV+ positive markers CD9/CD63/CD81 EVs. Statistical significance was reached for all lung and ovarian samples and for CD63+ EVs with gastric cancer. General lipid bilayer staining with CM did not show the significance that established EVs markers did. Results are presented as mean ± SEM, with statistical significance identified by Wilcoxon test and illustrated as *p<0.05, **p<0.01, ***p<0.001, ****p<0.0001.

Advancing from general/non-EVs specific quantification by NTA, flow cytometry was used to detect and quantify EVs based on their surface tetraspanin markers (CD9, CD63, and CD81). In addition, an attempt was made to assess total EVs in isolates using a cell membrane lipid stain (Cell Mask®), which was paired with a size standard (Rosetta beads). Thus, using both size calibration (particles smaller than 1000 nm), and the cell membrane stain, we quantified membranous particles within the collected fractions of EVs sizes. When assessing for established EVs markers, each of the tetraspanin-positive EVs populations was found in greater quantities in fractions collected from qEV35 compared to qEV70. Specifically, CD9+ EVs and CD81+ EVs collected with qEV35 versus qEV70 were at significantly greater quantities in isolates from both lung and ovarian cancer patients’ plasma (Fig. 4. A, E, C, G). With gastric cancer samples, quantities of both were also higher, but the differences were not statistically significant (Fig. 4. I and K). CD63+ EVs were at significantly greater quantities in qEV35 versus qEV70 isolates for all three cancer types (Fig. 4. B, F, J). Altogether, this indicates that regardless of the cancer type and the exact method of routine blood collection and plasma processing, qEV35 compared to qEV70 columns yield more tetraspanin-positive EVs. Interestingly, if simply assessing cell mask (CM+) objects as “total EVs particles”, no significant differences in quantities of EVs in isolates were found with qEV35 and qEV70, for any of the cancer types (Fig. 4. D, H, L).

**Fig. 5:**
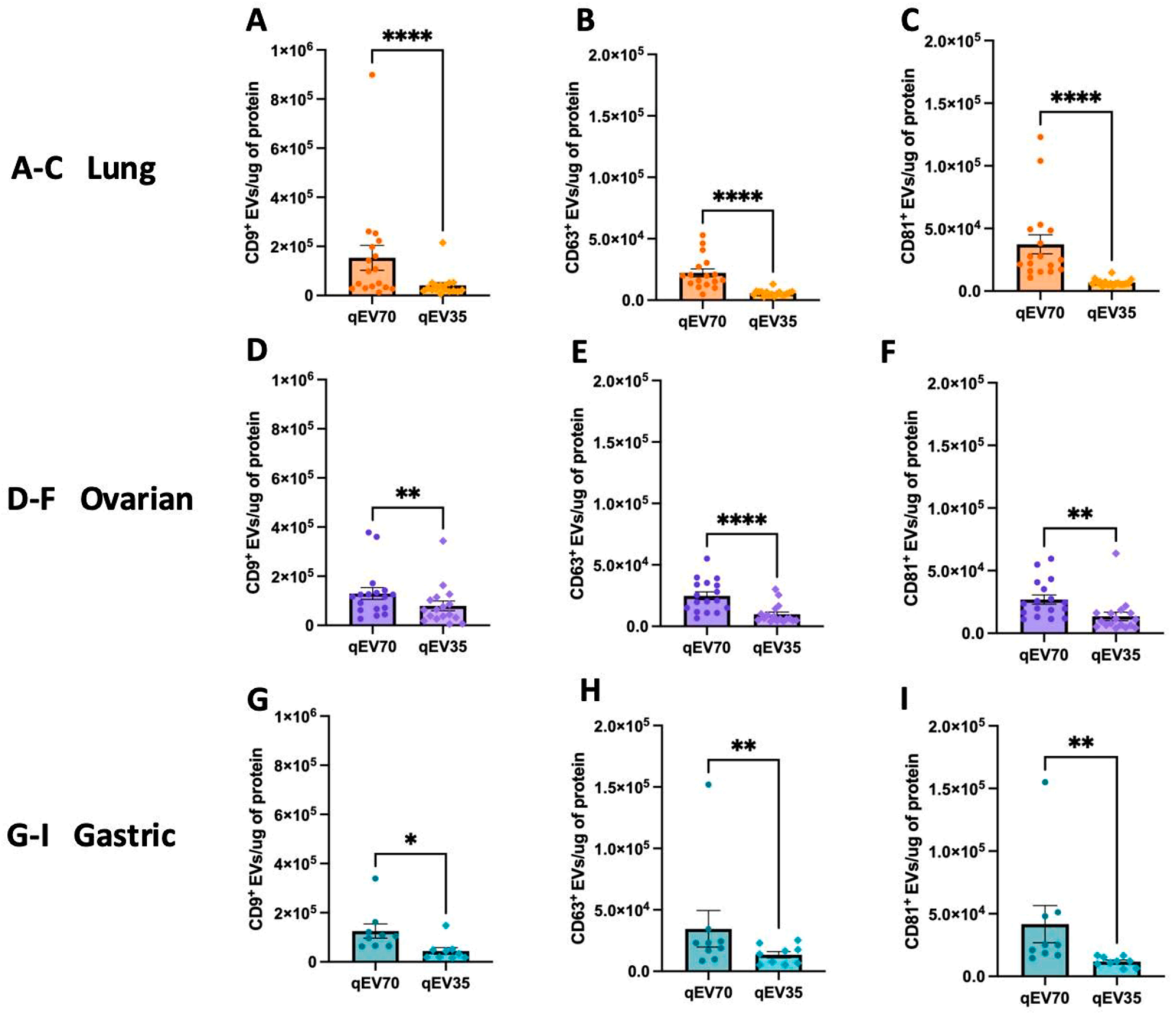
**Assessment of EVs purity, consider**ing **EVs surface markers.** A ratio was calculated to determine the comparative CD9+ EVs quantities in one ugof protein for isolates collected from (A) lung, (D) ovarian, and (G) gastric patients’ samples with qEV70 or qEV35 columns. A similar ratio was determined for CD63+ EVs (B, E, H), CD81+ EVs (C, F, I) in one ug of protein respectively (Mean ± SEM, Wilcoxon test). Significance expressed through p-value, *p<0.05, **p<0.01, ****p<0.0001.

Advancing on the purity estimate described by Webber and Clayton [21] which considers particle quantification by NTA/total protein, here we elected to also use our flow cytometric analysis of EVs quantities based on their surface tetraspanins CD9+/ CD63+/ CD81+ EVs per µg of protein. For all three cancer types the relative purity (i.e. quantities of CD9+/ CD63+/ CD81+ EVs/µg protein) was found to be significantly lower in qEV35 versus qEV70 isolates (Fig. 5A-I).

### Assessing if column usage increases contaminant amount

**Fig. 6:**
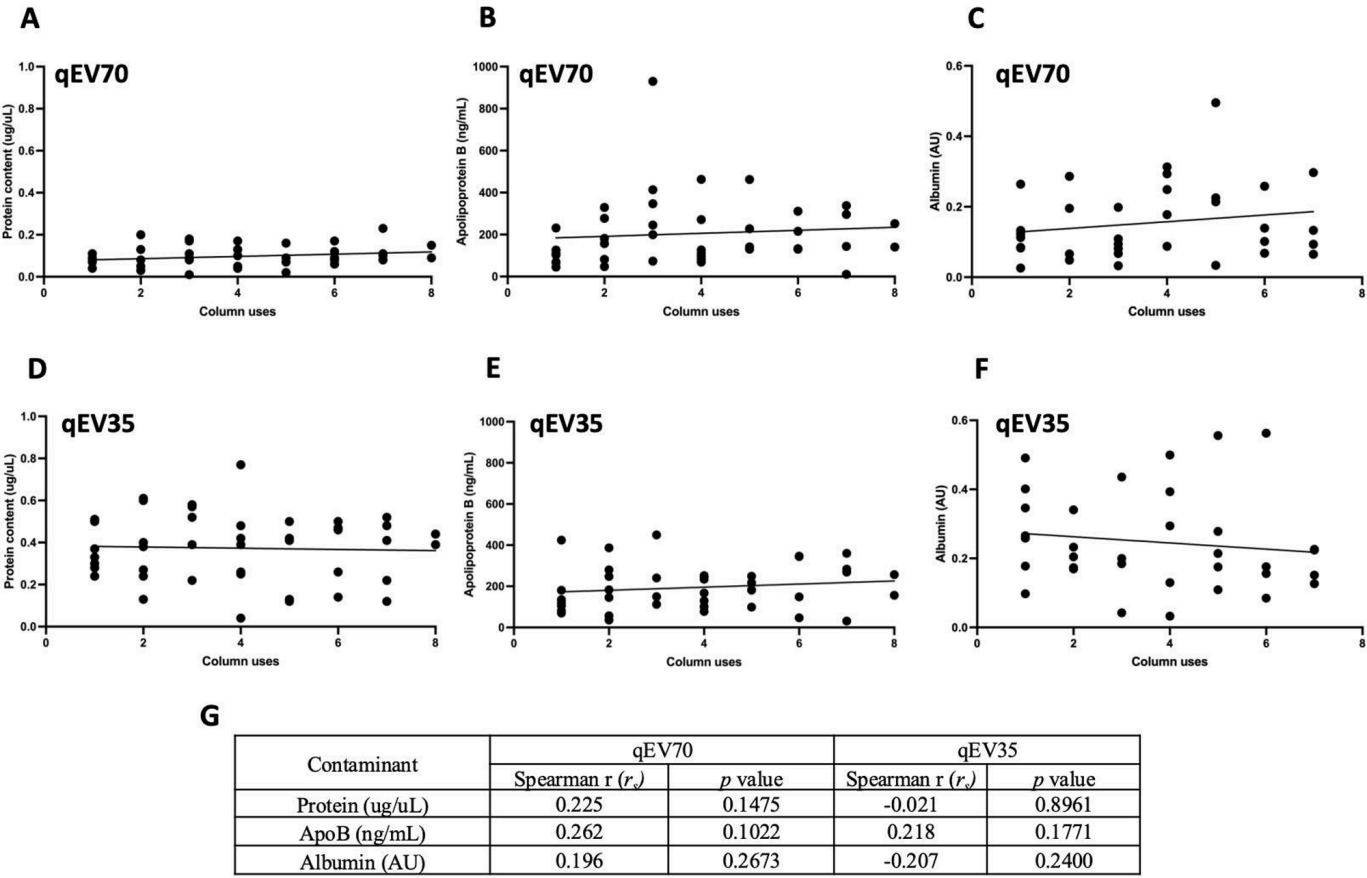
**Assessing re-usability of columns.** Contamination was assessed with repetitive column usage by plotting column uses (from 1st to 8th use) versus the amount of protein (**A**) in qEV70 and (**D**) qEV35 columns isolates. This was also performed for apolipoprotein B (**B**) qEV70 and (**E**) (qEV35), and albumin (**C**) qEV70 and (**F**) qEV35 to check if the amount of these contaminants increased with repetitive column use. Results are presented as the *Spearman r*, and *p* value for each correlation Table (**G**).

The barcode scan on the SEC used in this study enables the columns to be used up to ten times, but the manufacturers recommend use up to five times. Using pooled samples of plasma from healthy volunteers, other researchers have reported reduced purity between re-uses with a four-fold increase in Apo B after the second column use [18]. While that was an important study, it is noteworthy that the samples used were from non-fasting individuals, while those included here were from fasting cancer patients prior to medical intervention and so are of direct relevance to the intention of identifying a clinically applicable method for EVs collection as blood-based biomarkers identifies correlation with poorer outcome in a range of common cancer types. To establish what their re-use might be using individual cancer patients’ samples, we tested our qEV70 and qEV35 isolates eight times for total protein, ApoB and albumin (Fig. 6A-F). Interestingly, we observed no significant increase in any of these three parameters in our individual plasma sample isolates from patients, regardless of the cancer type cancer patient samples i.e. while intra-patients’ variability was understandably found between samples, a significant increase of a so-called contaminant with each successive column use was not seen.

### Correlations between quantities of plasma EVs, their potential as diagnostics biomarkers, and associations with patients clinicopathological details

Regarding diagnostic potential, given that CD63+ and CD81+ EVs appeared to be of particular interest in cancer, to subsequently consider their diagnostic relevance, we analysed the abundance of CD63+ EVs and CD81+ EVs in both qEV70 and qEV35 isolates from healthy donors too. As shown in Supplemental Fig. 4, regardless of the type of SEC used for their collection, CD63+ EVs were significantly more abundant in plasma from all cancer patients compared to that of healthy donors. CD81+ EVs collected using qEV35 columns were also significantly more abundant in plasma from all cancer patients compared to that of healthy donors, but following CD81+ EVs collection with qEV70 columns, significant differences were only found for lung cancer.

Supporting these observations, receiver operating characteristic (ROC) analysis showed that for all three cancer types, regardless of SEC-collection method, CD63+ EVs showed great discriminatory potential between healthy controls and patients with cancer (Supplemental Fig. 5). Of note, the discriminatory potential was enhanced when EVs were collected with qEV35 columns compared to the qEV70 columns (lung cancer AUC: 1.000, p < 0.0001 vs AUC:0.9235, p = 0.0003; ovarian cancer AUC: 1.000, p < 0.0001 vs AUC:0.9941, p < 0.0001; gastric cancer AUC: 1.000, p = 0.0002 vs AUC:0.9889, p = 0.0003). CD81+ EVs also showed discriminatory potential with statistical significance reached, for all cancer types, when EVs were collected with qEV35 columns (lung cancer AUC: 0.8529, p = 0.0026; ovarian cancer AUC: 0.8813, p = 0.0013; gastric cancer AUC: 0.7778, p = 0.0412). For qEV70 collected CD81+ EVs, showed statistically significant discriminatory potential was only seen for lung cancer (AUC: 0.7471, p = 0.035) (Supplemental Fig. 6). Taken together, our preliminary observations are that CD63+ EVs and CD81 EVs show the potential of distinguishing between healthy donors and patients with cancer, particularly when EVs are collected with qEV35 columns.

Correlations between quantities of plasma EVs and patients’ clinical information were also investigated, considering CD9+ EVs, CD63+ EVs, CD81+ EVs, and all TSPAN+ EVs (Table 1).

For ovarian cancer, we found a positive correlation between CD9+ EVs and patients’ age, for those >56 years old. This correlation was strong and significant for EVs collected using qEV70 columns (*r_s_*= 0.684, *p* = 0.0489), and very strong and significant for EVs collected with qEV35 columns (*r_s_*= 0.827, *p* = 0.0086). The correlation also held true when considering all TSPAN+ EVs. Here EVs collected with both columns showed very strong correlations with the patients aged >56y (qEV70 - *r_s_*= 0.819, *p* = 0.0097; qEV35 *r_s_*= 0.827, *p* = 0.009). This correlation indicates that for patients with ovarian cancer who are over the age of 56y, aging is associated with more CD9+ EVs and more TSPAN+ EVs.

For both lung and ovarian cancers (Tables 1A/1B), correlations were found between CD81+ EVs and progression free survival (PFS). Here the correlation was inverse, meaning higher quantities of plasma CD81+ EVs were associated with a shorter time to disease progression. Specifically, for lung cancer, the correlation was moderate (*r_s_*= −0.592, *p* = 0.014), and for ovarian cancer patients the correlation was strong (*r_s_*= −0.622, *p* = 0.009). Importantly, this was only true when assessing EVs collected with qEV70 columns.

**Table 1A:** Correlations between EVs and clinicopathological characteristics in ovarian cancer.

OVARIAN CANCER Clinical parameter	SEC		CD9^+^ EVs		CD63^+^ EVs		CD81^+^ EVs		All TSPAN^+^EVs	
Average	**qEV70**		1.06E+07 2.13E+07		2.09E+06 2.56E+06		2.35E+06 3.39E+06		1.51E+07 2.73E+07	
	**qEV35**									
		**Patients**	***Spearman r***	***p value***	***Spearman r***	***p value***	***Spearman r***	***p value***	***Spearman r***	***p value***
Age (median 56 years) <56	**qEV70**	**8/17**	−0.096	0.8247	−0.287	0.4844	−0.539	0.1765	−0.252	0.5473
	**qEV35**		0.012	0.9905	−0.120	0.7793	0.036	0.9434	−0.120	0.7793
Age (median 56 years) ≥56	**qEV70**	**9/17**	0.684	0.0489*	0.354	0.3472	0.165	0.6675	0.819	0.0097**
	**qEV35**		0.827	0.0086**	0.540	0.1372	0.118	0.7649	0.827	0.0086**
Smoker (never/ex-smoker/ current)	**qEV70**	**17/17**	0.037	0.9030	0.347	0.1788	0.111	0.6769	0.087	0.7530
	**qEV35**		0.334	0.1947	0.458	0.0698	0.210	0.4236	0.347	0.1788
PFS (Months) [Range 1–23]	**qEV70**	**17/17**	0.032	0.9036	−0.326	0.2002	−0.622	0.0090**	−0.220	0.3925
	**qEV35**		−0.166	0.5210	−0.363	0.1517	−0.447	0.0735	−0.137	0.5986
CA125 [Range 15–1977U/mL]	**qEV70**	**17/17**	0.025	0.9284	0.299	0.2430	0.302	0.2376	0.191	0.4609
	**qEV35**		0.169	0.5152	0.076	0.7729	0.176	0.4967	0.218	0.3988
CA125 [Range 15–1977U/mL] >35U/mL*	**qEV70**	**14/17**	−0.160	0.5838	0.121	0.6818	0.279	0.3305	−0.007	0.9879
	**qEV35**		0.174	0.5526	0.222	0.4448	0.068	0.8201	0.174	0.5526
Stage (I-IV)	**qEV70**	**17/17**	−0.253	0.3236	0.095	0.7136	0.470	0.0587	−0.024	0.9275
	**qEV35**		−0.085	0.7433	0.248	0.3336	0.450	0.0711	0.079	0.7620

⁎ 35U/mL – cut-off value for CA125

**Table 1B:** Correlations between EVs and clinicopathological characteristics in lung cancer.

LUNG CANCER Clinical parameter	SEC		CD9^+^ EVs		CD63^+^ EVs		CD81^+^ EVs		All TSPAN^+^EVs	
Average	**qEV70**		8.71E+06 1.58E+07		1.58E+06 2.34E+06		2.35E+06 2.79E+06		1.26E+07 2.09E+07	
	**qEV35**									
		**Patients**	***Spearman r***	***p value***	***Spearman r***	***p value***	***Spearman r***	***p value***	***Spearman r***	***p value***
Age (median 71 years) <71	**qEV70**	**8/17**	−0.381	0.3599	0.357	0.3894	−0.286	0.5008	−0.452	0.2675
	**qEV35**		−0.143	0.7520	0.286	0.5008	−0.595	0.1323	−0.190	0.6646
Age (median 71 years) ≥71	**qEV70**	**9/17**	−0.387	0.3044	0.555	0.1265	0.403	0.2820	−0.202	0.6022
	**qEV35**		−0.387	0.3044	0.605	0.0905	−0.185	0.6342	−0.252	0.5108
Sex (M/F)	**qEV70**	**17/17**	0.144	0.6058	0.337	0.1996	0.265	0.3213	0.168	0.5414
	**qEV35**		0.120	0.6730	0.192	0.4807	−0.241	0.3704	0.144	0.6058
Smoker (never/ ex-smoker/ current)	**qEV70**	**17/17**	−0.335	0.2353	0.261	0.3676	−0.075	0.8235	−0.298	0.2941
	**qEV35**		−0.037	0.9412	−0.075	0.8235	−0.335	0.2353	−0.037	0.9412
PFS (Months) [Range 3–22]	**qEV70**	**17/17**	−0.205	0.4278	−0.240	0.3499	−0.592	0.0138*	−0.258	0.3156
	**qEV35**		−0.237	0.3576	−0.559	0.0215*	−0.364	0.1510	−0.307	0.2291
Size [Range 11–100 mm]	**qEV70**	**17/17**	0.033	0.9002	−0.342	0.1779	0.271	0.2902	0.003	0.9944
	**qEV35**		−0.040	0.8777	−0.151	0.5605	0.353	0.1636	−0.016	0.9528
Stage (I-III)	**qEV70**	**17/17**	−0.120	0.6449	−0.195	0.4496	−0.046	0.8623	−0.210	0.4136
	**qEV35**		−0.257	0.3155	−0.333	0.1906	0.153	0.5532	−0.274	0.2839
Lymph node involvement (Y/N)	**qEV70**	**17/17**	0.096	0.7430	−0.217	0.4234	0.241	0.3704	0.000	>0.999
	**qEV35**		0.048	0.8884	−0.265	0.3213	0.120	0.6730	0.000	>0.999

In relation to PFS, a correlation was also observed between quantities of CD63+ EVs and PFS for the lung cancer patient cohort. However, this was observed for EVs collected with qEV35 only. Incidentally, this was also a moderate, inverse correlation (*r_s_*= −0.559, *p* = 0.022), meaning that lung cancer patients with greater quantities of plasma CD63+ EVs detected experienced a shorter time to disease progression.

For gastric cancer (Table 1C(i)), a correlation was found to exist between quantities of plasma CD9+ EVs and disease stage. This was a strong, positive correlation, meaning the more advanced the disease stage, the higher the CD9+ EVs counts. It is noteworthy that this was true for EVs collected with either SEC type (qEV70 - *r_s_*= 0.745, *p* = 0.028; qEV35 *r_s_*= 0.783, *p* = 0.016). Interestingly, TSPAN+ EVs collected with qEV35 also showed a very strong positive correlation with the patients’ disease stage (*r_s_*= 0.842, *p* = 0.008). Also, in the gastric cancer cohort, we found a very strong positive correlation between CD9+ EVs collected with qEV35 and N stage (involvement of lymph nodes by cancer - *r_s_*= 0.806, *p* = 0.013).

**Table 1Ci:** Correlations between EVs and clinicopathological characteristics in gastric cancer.

GASTRIC CANCER Clinical parameter	SEC		CD9^+^ EVs		CD63^+^ EVs		CD81^+^ EVs		All TSPAN^+^EVs	
Average	**qEV70**		8.71E+06 1.58E+07		1.58E+06 2.34E+06		2.35E+06 2.79E+06		1.26E+07 2.09E+07	
	**qEV35**									
		**Patients**	***Spearman r***	***p value***	***Spearman r***	***p value***	***Spearman r***	***p value***	***Spearman r***	***p value***
Age (median 64 years) <64	**qEV70**	**4/9**	0.400	0.7500	−0.200	>0.9167	−0.211	0.8333	−0.200	0.9167
	**qEV35**		−0.400	0.7500	0.200	0.9167	0.800	0.3333	0.800	0.3333
Age (median 64 years) ≥64	**qEV70**	**5/9**	−0.410	0.5000	−0.359	0.6333	0.667	0.2667	−0.410	0.5000
	**qEV35**		0.103	0.9000	−0.462	0.4333	0.667	0.2667	0.103	0.9000
Sex (M/F)	**qEV70**	**9/9**	0.365	0.3810	−0.046	0.9643	−0.229	0.5952	0.456	0.2619
	**qEV35**		0.365	0.3810	−0.365	0.3810	−0.091	0.9048	0.183	0.6667
Smoker (never/ ex-smoker/ current)	**qEV70**	**9/9**	−0.456	0.2381	0.046	0.9397	0.413	0.2857	−0.365	0.3587
	**qEV35**		−0.456	0.2381	0.091	0.8603	0.274	0.4984	−0.183	0.6603
Tumour regression grade [3-5]	**qEV70**	**7/9**	−0.231	0.6714	−0.545	0.2286	0.694	0.1286	−0.154	0.7857
	**qEV35**		−0.077	0.9571	−0.154	0.7857	0.386	0.4286	0.000	>0.999
Clinical stage (II-IV)	**qEV70**	**9/9**	0.745	0.0278*	−0.187	0.6468	−0.187	0.6389	0.596	0.1071
	**qEV35**		0.783	0.0159*	0.559	0.1270	0.075	0.8730	0.842	0.0079**
Clinical T stage [2-4]	**qEV70**	**9/9**	0.274	0.5833	−0.367	0.3889	−0.321	0.4444	0.273	0.5833
	**qEV35**		0.274	0.5833	−0.091	>0.999	−0.183	0.7778	0.092	0.9722
Clinical N stage (0–3)	**qEV70**	**9/9**	0.511	0.1643	−0.422	0.2496	0.087	0.8248	0.451	0.2222
	**qEV35**		0.806	0.0127*	0.312	0.4087	0.017	0.9714	0.679	0.0508

Given the small cohort sizes, particularly for gastric cancer, multiple testing correction was applied in the forms of a Holm-Šidák correction. Following this adjustment, statistical significance was then lost as indicated in Table 1C(ii).

**Table 1 Cii:** Correlations between EVs and clinicopathological characteristics in gastric cancer, following multiple testing correction due to small cohort size.

GASTRIC CANCER Clinical parameter	SEC		CD9^+^ EVs		CD63^+^ EVs		CD81^+^ EVs		All TSPAN^+^EVs	
Average	**qEV70**		8.57E+06 1.10E+07		1.77E+06 2.87E+06		2.31E+06 2.82E+06		1.27E+07 1.67E+07	
	**qEV35**									
		**Patients**	***Spearman r***	***p value***	***Spearman r***	***p value***	***Spearman r***	***p value***	***Spearman r***	***p value***
Age (median 64 years) <64	**qEV70**	**4/9**	0.400	1.000	−0.200	1.000	−0.211	1.000	−0.200	1.000
	**qEV35**		−0.400	1.000	0.200	1.000	0.800	0.961	0.800	0.961
Age (median 64 years) ≥64	**qEV70**	**5/9**	−0.410	0.969	−0.359	0.969	0.667	0.916	−0.410	0.969
	**qEV35**		0.103	0.990	−0.462	0.967	0.667	0.916	0.103	0.990
Sex (M/F)	**qEV70**	**9/9**	0.365	0.965	−0.046	0.991	−0.229	0.973	0.456	0.912
	**qEV35**		0.365	0.965	−0.365	0.965	−0.091	0.991	0.183	0.973
Smoker (never/ex-smoker/current)	**qEV70**	9/9	−0.456	0.886	0.046	0.980	0.413	0.886	−0.365	0.892
	**qEV35**		−0.456	0.886	0.091	0.980	0.274	0.937	−0.183	0.961
Tumour regression grade [3-5]	**qEV70**	**7/9**	−0.231	0.996	−0.545	0.837	0.694	0.667	−0.154	0.968
	**qEV35**		−0.077	0.998	−0.154	0.998	0.386	0.965	0.000	1.000
Clinical stage (II-IV)	**qEV70**	**9/9**	0.745	0.156	−0.187	0.953	−0.187	0.953	0.596	0.433
	**qEV35**		0.783	0.106	0.559	0.433	0.075	0.953	0.842	0.062
Clinical T stage [2-4]	**qEV70**	**9/9**	0.274	0.995	−0.367	0.981	−0.321	0.984	0.273	0.995
	**qEV35**		0.274	0.995	−0.091	1.000	−0.183	0.995	0.092	0.999
Clinical N stage (0–3)	**qEV70**	**9/9**	0.511	0.659	−0.422	0.715	0.087	0.969	0.451	0.715
	**qEV35**		0.806	0.097	0.312	0.793	0.017	0.971	0.679	0.306

In lung cancer and ovarian cancer where correlations were observed with PFS, Kaplan-Meier curves and Cox regression models were applied to establish if their outcomes would support EVs having prognostic (as well as diagnostic) potential. In ovarian cancer, qEV70 collected CD81+ EVs correlation (p = 0.009) described above was supported by Cox regression, showing a hazard ratio of 2.006. While statistical significance was not reached (p = 0.082) with the associated Kaplan-Meier curve, its trend was in agreement (Supplemental Fig. 7). In relation to lung cancer, while the qEV70 collected CD81+ EVs correlation described above (p = 0.0138) was not supported by Cox regression or the Kaplan-Meier curve, the qEV35 collected CD63+ EVs correlation (p = 0.0215) was supported by Cox regression (hazard ratio = 5.008), and the associated Kaplan-Meier curve trend was in agreement (0.0897) (Supplemental Fig. 7).

## Discussion

Extracellular vesicles are gaining substantial interest from patient groups, clinicians, industry, and academic researchers as having substantial potential as sources of minimally invasive blood-based liquid biopsies. While there are some high-quality published papers supporting this, frequently those studies involve analysis of relatively large volumes of blood plasma or serum (e.g. 3–5 mL) obtained by pooling healthy donors’ samples, as proof of principle. Here, with the focus on truly bridging EVs laboratory research toward the clinical setting for the benefit of patients with cancer who are already undergoing many blood draws for other essential tests (e.g. monitoring of white blood cell counts) we elected to critically evaluate SEC for this setting. For this reason, we considered “real world” plasma samples from a range of common cancer types that are too often diagnosed late and so where there is a real unmet clinical need.

SEC columns can be prepared in-house or purchased, and our team has experience of both approaches. However, as commercial columns are typically packed under strictly controlled conditions and may allow more reproducible results than home-made columns [13], we elected to use different offering of the latter i.e. qEV70 and qEV35 columns. SEC is considered a relatively quick (possibly the quickest) method for EVs collection; is not biased on pre-determined EVs surface markers to isolate only particular sub-populations of EVs; does not require expensive equipment; and is not difficult to use. Despite the benefits of SEC, given that it is a technique for size-based separation of particles, other non-EVs particles, such as lipoproteins and protein complexes – described as “contaminants” - may co-migrate, if present in the starting samples and dependent on the matrix composition and pore sizes of the SEC column [13]. Of note, while research suggests that such so called contaminants (ApoB, [22]) may be part of the natural protein corona which associates with EVs once they are released by cells into the extracellular milieu and may be necessary for EVs functionality e.g. facilitating increased or decreased cellular uptake [23], for the purpose of this study focused on EVs as blood-based biomarkers, we gave consideration to such co-migrants (albumin and ApoB) as contaminants.

Considering the time needed to collect EVs, we found that the steps of processing each of the n = 53 plasma samples through qEV70 SEC took approximately 10–15mins, and approximately 20+ mins for qEV35 SEC. Although the manufacturers indicate qEV35 to be optimal for enrichment of 35–350 nm particles and qEV70 for 70–1000 nm, as the EVs in the samples for all three cancer types averaged 150–200 nm in size in these “real world” samples as assessed by nanoparticle tracking analysis, which SEC column was used was irrelevant in that regard. Similarly, transmission electron microscopy analyses of particles structures and immunoblotting analyses for EVs positive and negative markers were supportive of the collected particles predominant being EVs. Interestingly, for all cancer types, regardless of how the blood plasma was pre-processed (specific to the cancer type biobanking SOPs), NTA reported significantly more particles retrieved with qEV35 compared to qEV70 columns. This was in keeping with a report from a small study of n = 6 prostate cancer patients’ plasma [16]. Investigating this further and considering albumin (an abundant protein in blood) as representative of potentially co-migrating proteins, and Apo B (a main contaminant in EVs samples separated from plasma [19]) as representative of potentially co-migrating lipoproteins, the qEV35 isolates were found to contain significantly more non-EV protein compared to the qEV70 isolates. Remarkably, no significant difference existed in Apo B co-migration between qEV35 and qEV70 isolates. Interestingly, this held true for all cancer types tested, regardless of their specific plasma pre-processing procedure.

Given that the main EVs co-migrating contaminant seemed to be protein, we progressed to applying the commonly used estimation of vesicles purity as described by Webber and Clayton, which considers particles concentration obtained by NTA and total protein concentration [21]. For all three cancer types, the total protein in our isolates was significantly greater following qEV35 versus qEV70. The particle amounts were also significantly greater following qEV35 versus qEV70, and only the lung cancer samples showed significantly reduced purity from qEV35 compared to qEV70 columns.

Although NTA is a commonly available platform in EVs research laboratories, as we highlighted in MISEV2023, the use of NTA to measure concentration of complex biofluids should be interpreted with caution due to counting of co-isolated non-EVs of similar sizes [13]. Flow cytometry is a more sophisticated but complex method for EVs analysis. Its use of antibodies specific for tetraspanins, such as CD9, CD63, CD81, which are considered to be surface hallmarks of EVs [13], affords more confidence that we are evaluating EVs. However, as some studies use a simpler approach of a general lipid membrane stain with flow cytometry as a surrogate for a specific surface member marker, we also considered this. Our results with the lipid membrane stain alone suggested EVs recovery were similar from qEV35 and qEV70 columns, for all cancer types. However, when we assessed EVs tetraspanin markers (CD9, CD63 and CD81), evidence suggested that significant increased EVs recovery was typical with qEV35 compared to qEV70 across each cancer types. The exceptions to this were gastric cancer where although qEV35 always resulted in greater EVs yields, statistical significance was only reached for CD63+ EVs. Of note, given that significantly more CD63+ EVs were found in cancer whether the EVs were collected with qEV70 or qEV35 SEC; that significantly more CD81+ EVs were found in cancer when the EVs were collected with qEV35 SEC; and that significantly more CD81+ EVs were found in lung cancer when the EVs were collected with qEV70 SEC are preliminary observations supporting EVs abundance having biomarker potential. Larger studies of all cancer cohorts, with matched samples from healthy donors are warranted to investigate this further.

With flow cytometry data generated, we advanced on the method described by Webber and Clayton [17] for EVs purity estimates. Specifically, instead of considering NTA-derived particles quantities to protein concentration, we considered CD9+, CD63+ and CD81+ quantification by flow cytometry to protein concentration. Applying the more EVs-specific approach of flow cytometry, qEV35 isolates were always deemed significantly less pure than qEV70 isolates. Although using flow cytometry allows us to selectively analyse EVs, not albumin, due to EVs surface CD9+, CD63+ and/or CD81+ content (which is missing from albumin), consideration should be given to the presence/possible presence of albumin when performing downstream analysis that does not involve evaluation of a characteristic that distinguishes EVs from other matter, such as albumin. Of course, depending on the nature of future studies, consideration needs to be given as to whether efforts should be made to maintain the protein corona (including ApoB and albumin) due to their involvement in EVs functionality, or remove it [24], if considered EVs contaminants. However, even more directly related to this study, if a decision was made to further analyse the EVs in the clinical setting using simple techniques such as an ELISA where a fixed concentration of isolate would normally be used, our evidence suggests that the likelihood of analysing purer EVs rather than non-vesicular proteins would be higher with qEV70 isolates compared to qEV35 isolates.

MISEV2023 [13] notes that, in some cases, SEC matrices can be re-used after thorough cleaning. The qEV70 and qEV35 manufacturer recommends a maximum of five uses per column, although previous researchers analysing pooled samples of plasma from healthy volunteers found reduced purity between re-uses with a four-fold increase in Apo B after the second column use. Our testing of this feature included eight successful uses with a thorough cleaning and regeneration step between each use. The results showed no significant increase in total protein, ApoB and albumin in our individual plasma sample isolates from cancer patients, regardless of the cancer type or how the plasma was pre-processed. This difference in re-use of SEC with healthy volunteer pooled plasma and cancer patient individual plasma samples may warrant further investigation in a paired study.

Considering our strong interest in EVs as liquid biopsies, we were enthusiastic to also explore the biomarker potential of EVs. Our research showed that regardless of the blood collection and pre-processing method, SEC type used for EVs collection, and the cancer type or EVs collection method, CD63+ EVs (and to a lesser extent) CD81+ EVs abundance are preliminary observations supporting their potential as diagnostic biomarkers. However, it should be considered that, the use of different anticoagulants and filtration procedures between cohorts in pre-processing could potentially introduce confounding effects on EVs yield and composition. This may be particularly relevant to consider, depending on the down-stream processing intended for a given future study.

In conclusion, SEC for EVs collection from plasma has many attributes which suggest that, as a methodology, it can bridge EVs laboratory research to the clinical setting, once its limitations are also considered. While there is reduction in purity of EVs in qEV35 isolates versus the qEV70 isolates, with both SEC types relatively large quantities of EVs were successfully collected from small clinically-relevant plasma volumes (1 mL), regardless of how the plasma was pre-processed, and for all three cancer types. Of all 53 individual plasma samples, where EVs were collected from identical aliquots using qEV70 or qEV35 (i.e. a total of >100 isolations were performed), all samples delivered EVs that could be characterised in accordance with our MISEV2023 guidelines. Interestingly, there were no significant differences between the qEV70 and qEV35 isolates in terms of particle sizes, structure, and EVs positive and negative marker detection. Of particular note, the results generated in this study suggest that SEC has potential in the clinical setting as a means of collecting EVs for application of their abundance as diagnostic and prognostic biomarkers. Finally, while our proof-of-principle data suggest that SEC can be re-used multiple (at least eight) times, re-use would not be a suitable approach for a clinical setting.

## Funding

We wish to acknowledge the funding support as the All Ireland Cancer Liquid Biopsies Consortium (CLuB), which is a Strand II project funded under the North South Research Programme (NSRP). The NSRP is a collaborative scheme funded through the Government’s Shared Island Fund. It is being administered by the Higher Education Authority (HEA) on behalf of the Department of Further and Higher Education, Research, Innovation and Science. Also, the BOOST Award from Trinity College Dublin.

## Data availability

The raw data is available in Supplementary Material and from the corresponding author.

## Declarations

### Ethics approval and consent to participate

St James’s Hospital (SJH)/Tallaght University Hospital Joint Research Ethics Committee and in compliance with the General Data Protection Regulation (GDPR) and Data Protection Act 2018. Written informed consent was obtained from all patients prior to participation.

## Generative AI use

Gen AI was not used in any part of this research or in preparing this manuscript.

## CRediT authorship contribution statement

**Szilárd-Krisztián Belényesi:** Writing – review & editing, Writing – original draft, Methodology, Investigation, Formal analysis, Data curation. **Seán Patmore:** Writing – review & editing, Methodology, Investigation, Formal analysis, Data curation. **Sinead Hurley:** Writing – review & editing, Methodology. **Ezgi Oner:** Writing – review & editing, Methodology. **Volga M. Saini:** Writing – review & editing, Methodology. **Marika Kanjuga:** Writing – review & editing, Resources. **Faye Lewis:** Writing – review & editing, Resources. **Meghana S. Menon:** Writing – review & editing, Resources. **Christina Cahill:** Writing – review & editing, Methodology. **Antonia Tierney:** Writing – review & editing, Resources. **Oana Deac:** Writing – review & editing, Resources. **Maria Jose Santos Martinez:** Methodology, Resources, Writing – review & editing. **Maeve A. Lowery:** Writing – review & editing, Methodology. **John J. O’Leary:** Writing – review & editing, Resources. **Kathy Gately:** Writing – review & editing, Resources. **Sharon O’Toole:** Writing – review & editing, Resources. **Lorraine O’Driscoll:** Writing – review & editing, Resources, Project administration, Investigation, Funding acquisition, Formal analysis, Conceptualization.

## Declaration of competing interest

The authors declare that they have no known competing financial interests or personal relationships that could have appeared to influence the work reported in this paper.

## Appendix Supplementary materials

Image, application 1
