# Supplementary material for: Size-exclusion chromatography as a clinically applicable method for extracellular vesicles collection as liquid biopsies shows potential diagnostic relevance and correlation with poorer outcome in common cancer types
Source: Transl Oncol. 2026 Aug 4;72:102964. doi: 10.1016/j.tranon.2026.102964 (PMC13470115; doi:10.1016/j.tranon.2026.102964)
Supplement: Supplementary file 1 [file mmc1.pdf]

Supplementary Material

Supplemental Method

As described in the accompanying main manuscript, paired plasma samples were processed through both qEV70 and qEV35 SEC, and flow cytometry was used to assess the CD9+, CD63+ and CD81+ EVs.

Supplemental Method, part A+B (below): depicts the experimental steps that were performed to determine optimal gain conditions for the Cytek Aurora flow cytometer. Double filtered PBS and 150nm polystyrene beads were evaluated with progressively higher gains (500-4000) on the fluorescence channel (B2-A -488nm blue laser). The SSC-H value was then calculated for each run and median ± standard deviation values were plotted between background noise (PBS only) and polystyrene beads. Increased separation (quantified as separation index SI) was observed as gain is increased until 2000 gain, which marked a plateau in separation. 2000 gain represents maximal separation between sample detection and minimal excitation of background noise. Therefore, 2000 gain was used in all subsequent experiments, with re-calibration checks after engineer visits.

Supplemental Method, part (C): to prevent swarm detection, a serial dilution test was run on the qEV35 and qEV70 processed samples. Dilution of qEV35 and qEV70 samples was performed with a dilution series as follows: 10, 31.6, 100, 316.2, 1000, 3162, 10000, 31620, 100000, 316200 fold. A point of linearity was determined starting between the 31.6-100fold dilution, continuing until the 10,000-fold dilution. This marked the dilution range that EVs collected by qEV35 and qEV70 columns could be linearly measured via Cytek Aurora flow cytometer. Therefore, all subsequent experiments were performed using a 1/100 final dilution when measuring by flow cytometry, to prevent swarming, but maximising detection of positive EVs.

Supplemental Method, part (D): shows that size was tracked via Rosetta beads and calibrated using the Rosetta calibration software (Exometry), particles were considered EVs when less than 1000nm and containing EVs tetraspanin marker (CD9, CD63 or CD81). Flow rates were determined using Apogee bead counts. Apogee beads are packaged with known particle concentration, therefore by calculating total events we could determine the flow rate of each experiment ((total beads measured/ expected bead concentration)/ Time (min)). Flow rate was calculated in each new experiment to account for day-to-day variations in flow speed.

Supplemental Method, part (E): outlines the gating strategy applied to detect positive signal of EVs markers. Background noise was established using PBS, reagent controls and NP40 controls. Positive EVs were identified by gating the region beyond the background noise. The Rosetta bead FlowJo plugin was used to identify particles less than 1000nm gated for final enumeration. Events were considered EVs if they exhibited positive staining of tetraspanin markers and were less than 1000nm in size.

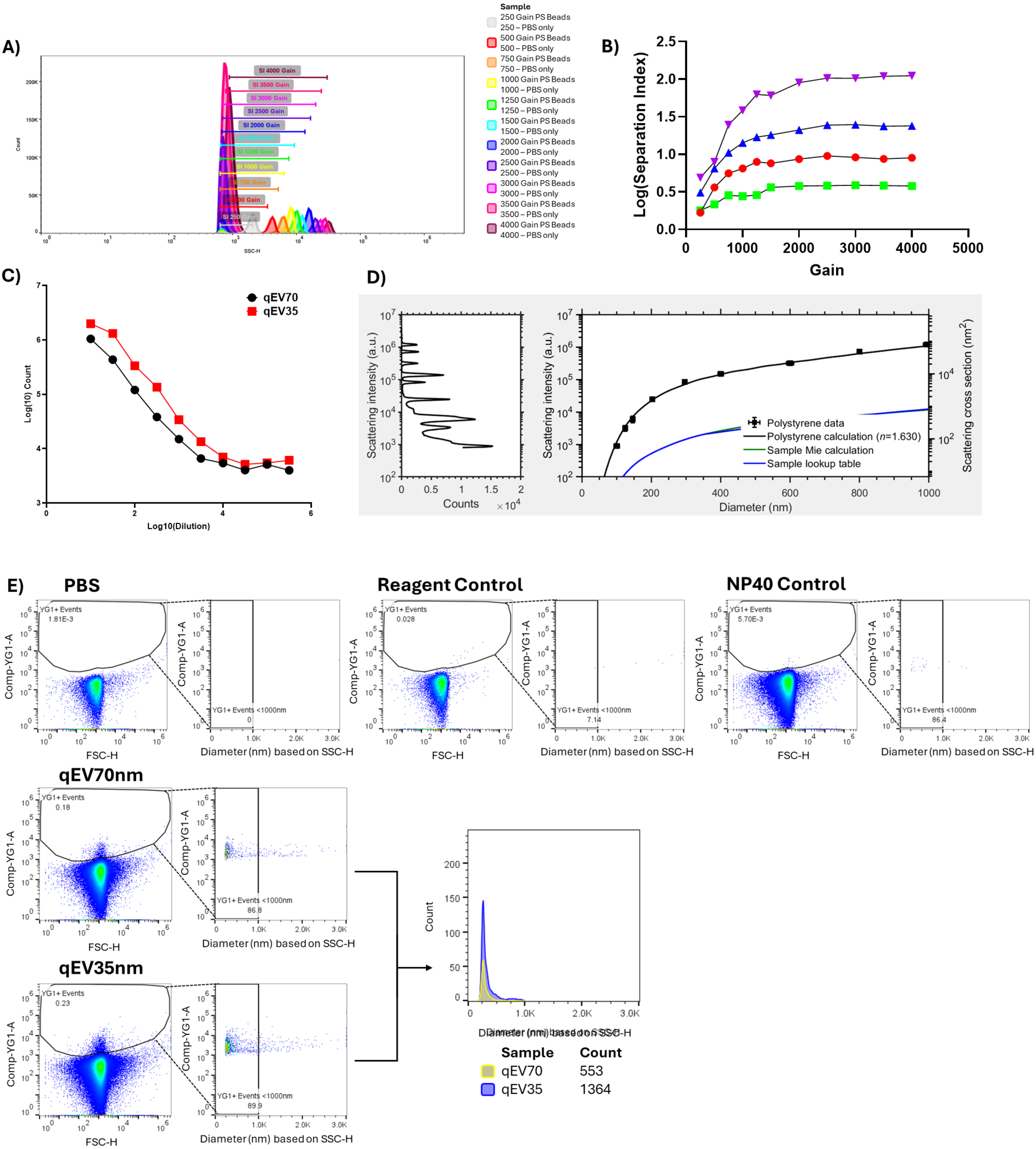
A)
B)
C)
D)
E)
Sample
250 Gain PS Beads
250 – PBS only
500 Gain PS Beads
500 – PBS only
750 Gain PS Beads
750 – PBS only
1000 Gain PS Beads
1000 – PBS only
1250 Gain PS Beads
1250 – PBS only
1500 Gain PS Beads
1500 – PBS only
2000 Gain PS Beads
2000 – PBS only
2500 Gain PS Beads
2500 – PBS only
3000 Gain PS Beads
3000 – PBS only
3500 Gain PS Beads
3500 – PBS only
4000 Gain PS Beads
4000 – PBS only
Log(Separation Index)
Gain
qEV70
qEV35
Log(10) Count
Log10(Dilution)
Scattering intensity (a.u.)
Counts
Polystyrene data
Polystyrene calculation (n=1.630)
Sample Mie calculation
Sample lookup table
Diameter (nm)
Scattering cross section (nm2)
PBS
Reagent Control
NP40 Control
qEV70nm
qEV35nm
Comp-YG1-A
FSC-H
Diameter (nm) based on SSC-H
YG1+ Events
YG1+ Events <1000nm
Count
Sample Count
qEV70 553
qEV35 1364

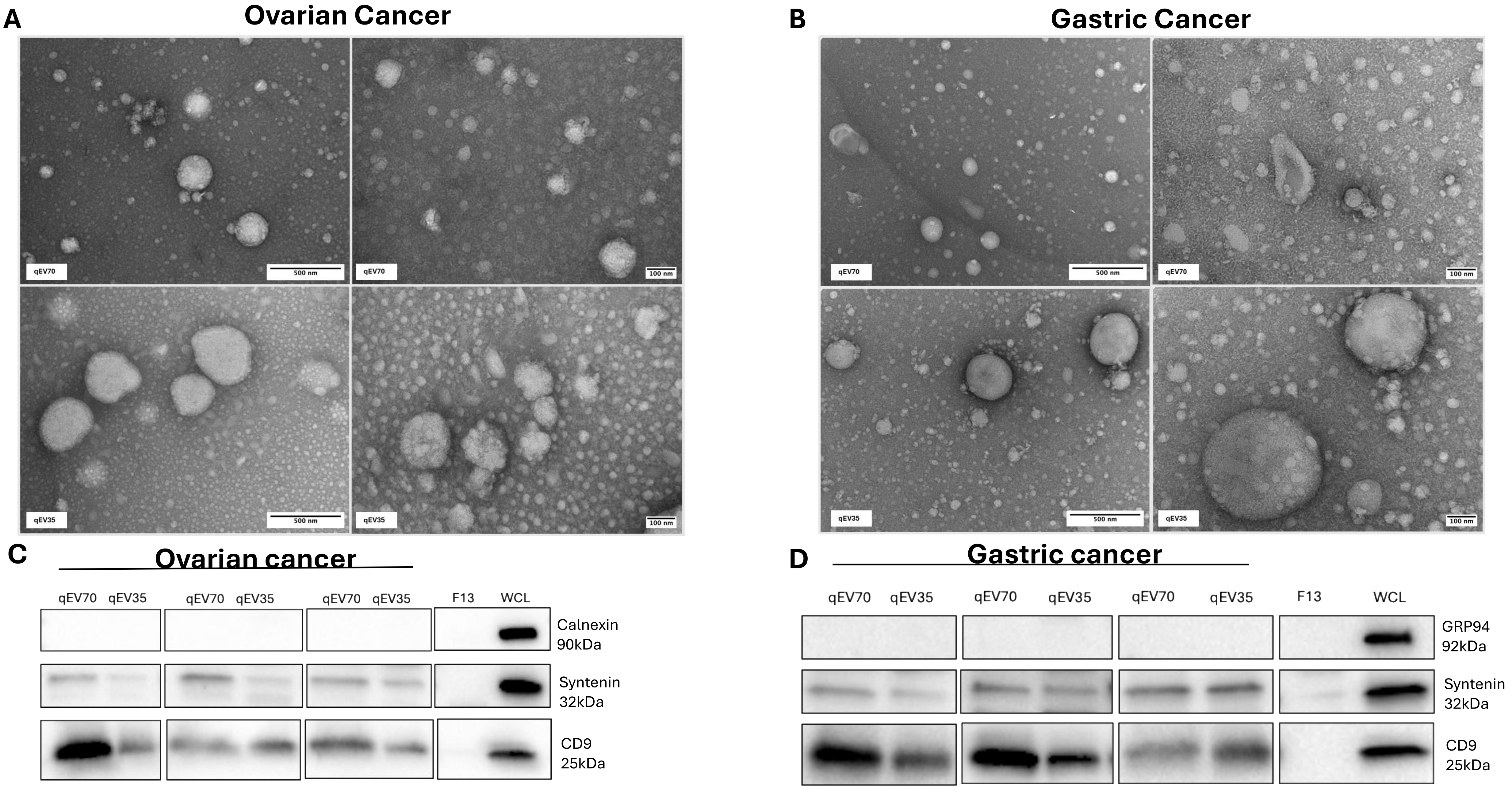

Supplemental Fig. 1. Characterisation of EVs (qEV70 and qEV35) from three cancer patient cohorts. Representative transmission electron microscopy (TEM) images of EVs from patients with cancer – images obtained at 5,000x and 30,000x for one patient with ovarian (**A**) and gastric (**B**) cancer. Immunoblot analysis showing detection of positive EVs markers (CD9, Syntenin), and lack of detection of EVs negative markers Calnexin or GRP94. WCL (whole cell lysate) was used as a control to demonstrate the presence of GRP94. These markers were probed for in EVs collected from all patients. (**C**) and (**D**) show representative image for n=3 ovarian and gastric cancer patients respectively.

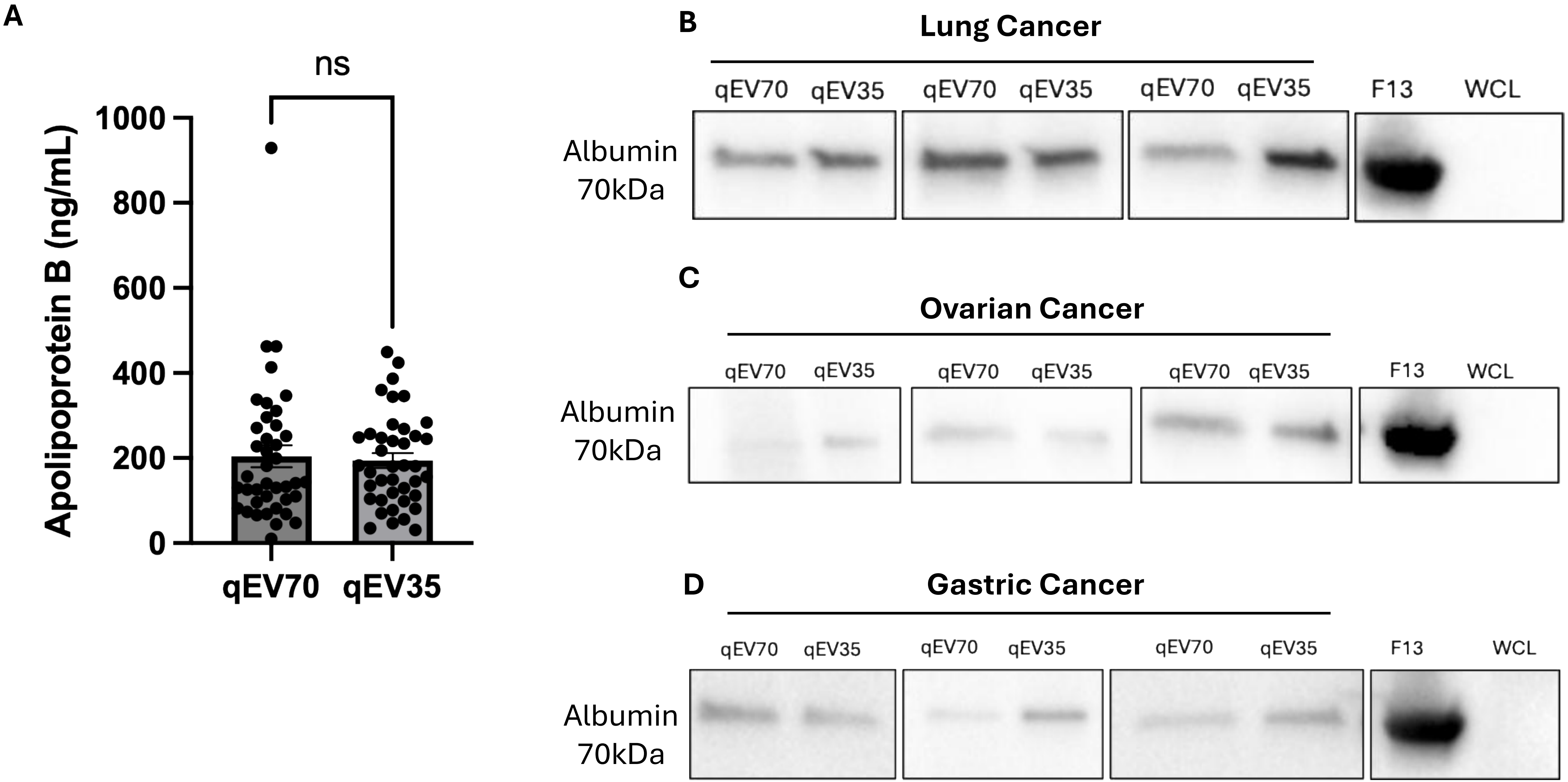

Supplemental Fig. 2. Assessment of lipoprotein and albumin with EVs collected from three cancer patient cohorts, using two different SEC (qEV70 and qEV35) columns. Comparison between Apolipoprotein B values in EV isolates from all patients samples included in this study (n=43) following collection by qEV70 or qEV35 columns (A). Representative immunoblotting images showing the presence of albumin in EVs isolates from n=3 patients with lung (B), ovarian (C), and gastric (D) cancer, compared to fraction 13 (F13) and whole cell lysate (WCL).

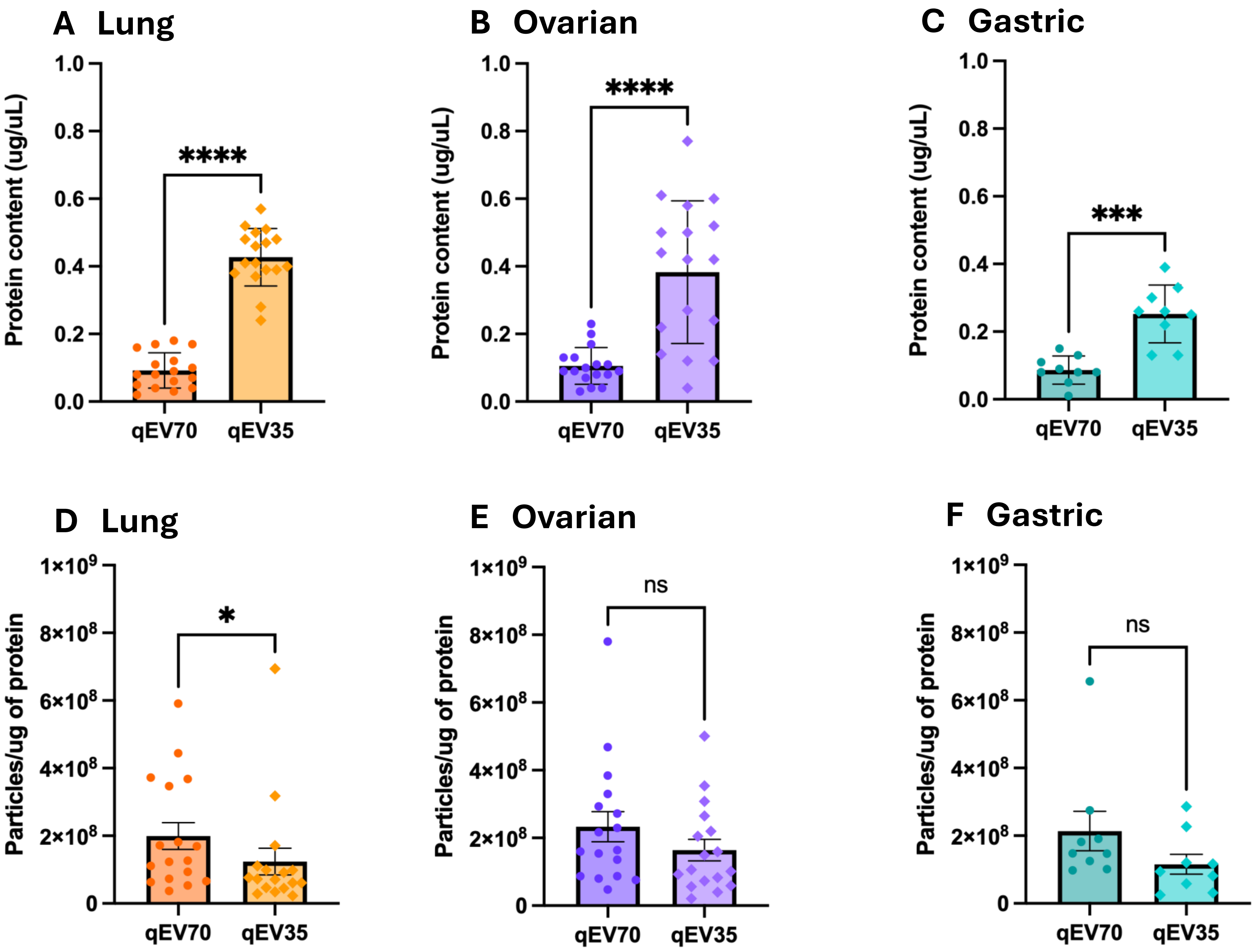

Supplemental Fig. 3. **Determining qEVs isolates purity**. Bradford assay showed significantly higher concentrations of protein collected in EVs isolates using qEV35 compared to qEV70, for each of (A) lung, (B) ovarian, and (C) gastric cancers (Mean ± SD, paired t-test). Using particle counts (detected by NTA) normalised to protein, a ratio was generated to represent the 'purity ratio' of EVs collected with qEV35 and qEV70 columns from plasma of patients with (D) lung, (E) ovarian, and (F) gastric cancer (Mean ± SEM, Wilcoxon test). Statistical significance is expressed as *$p<0.05$, **$p<0.01$, ***$p<0.001$, ****$p<0.0001$.

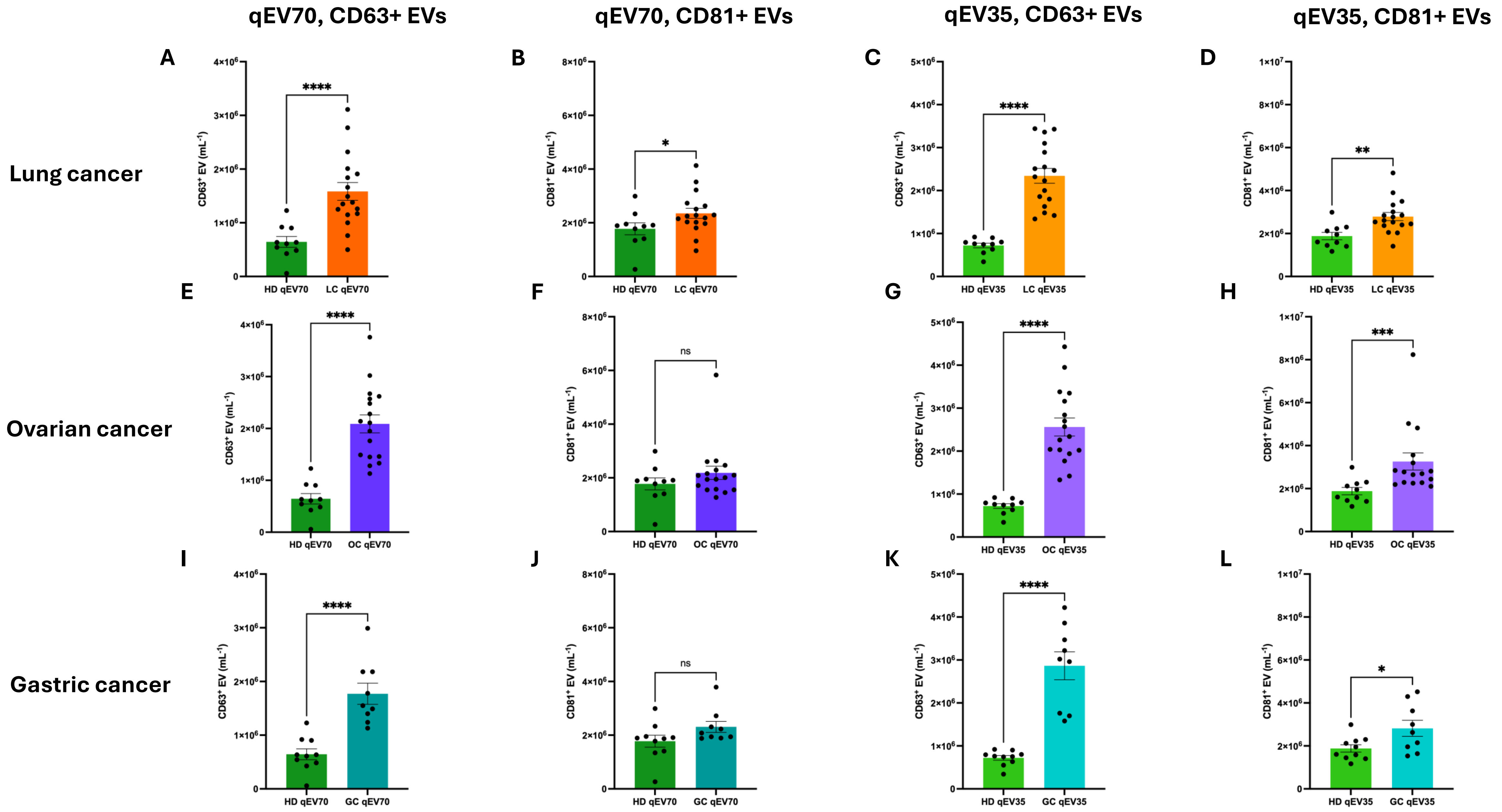

Supplemental Fig. 4. Potential diagnostic relevance of CD63+ EVs and CD81+ EVs. Considering lung (A-D, n=17), ovarian (E-H, n=17), and gastric cancer (I-L, n=9) samples compared to those from healthy controls (n=10), flow cytometry data typically showed significantly more CD63+ EVs and CD81+ EVs in cancer whether the EVs were collected with qEV70 or qEV35 SEC. The exceptions to this are CD81+ EVs collected by qEV70 in ovarian and gastric cancers. (Mean ± SEM, Mann-Whitney test). Statistical significance is expressed as *p<0.05, **p<0.01, ***p<0.001, ****p<0.0001.

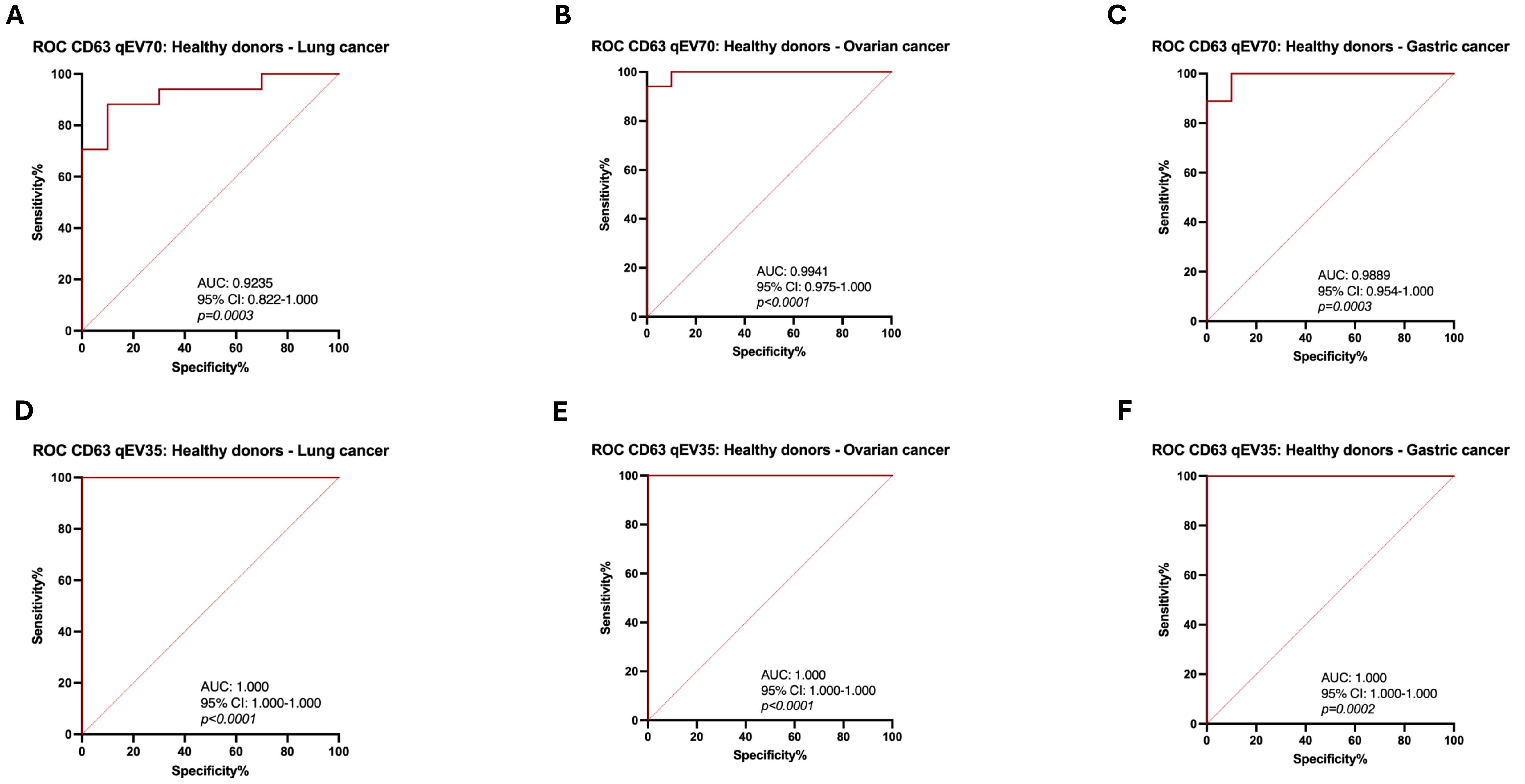

Supplemental Fig. 5. ROC analysis of CD63+ EVs in samples collected from lung, ovarian, and gastric cancer plasma and healthy donor plasma with qEV70 and qEV35. ROC analysis determined the discriminatory potential of CD63+ EVs by comparing the abundance of CD63+ EVs in samples from healthy donors with those from patients with lung (**A** – qEV70, **D** – qEV35), ovarian (**B** – qEV70, **E** – qEV35), and gastric (**C** – qEV70, **F** – qEV35) cancers.

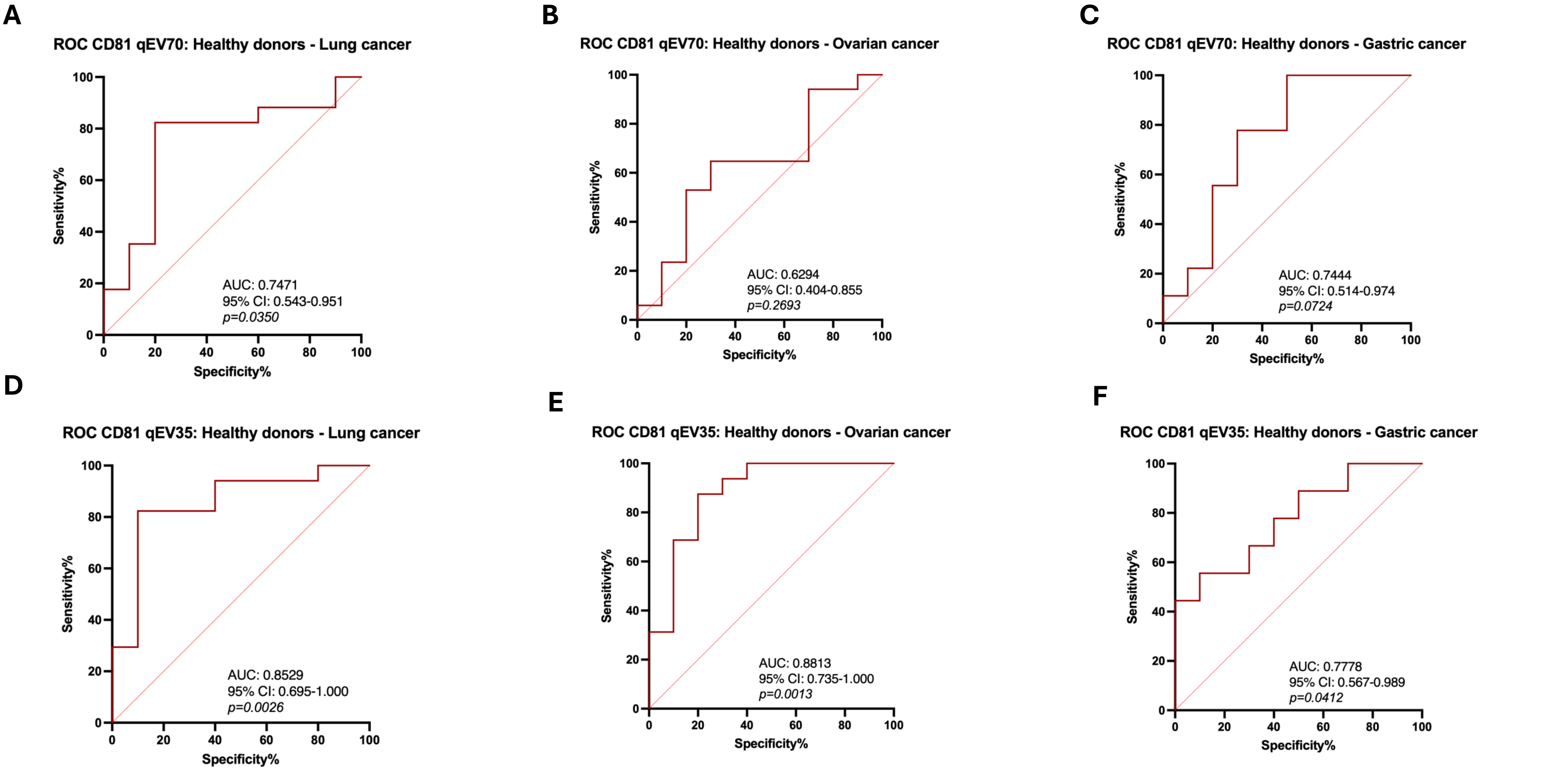

Supplemental Fig. 6. ROC analysis of CD81+ EVs in samples collected from lung, ovarian, and gastric cancer plasma and healthy donor plasma with qEV70 and qEV35. ROC analysis determined the discriminatory potential of CD81+ EVs by comparing the abundance of CD81+ EVs in samples from healthy donors with those from patients with lung (**A** – qEV70, **D** – qEV35), ovarian (**B** – qEV70, **E** – qEV35), and gastric (**C** – qEV70, **F** – qEV35) cancers.

Kaplan Meier: Lung cancer

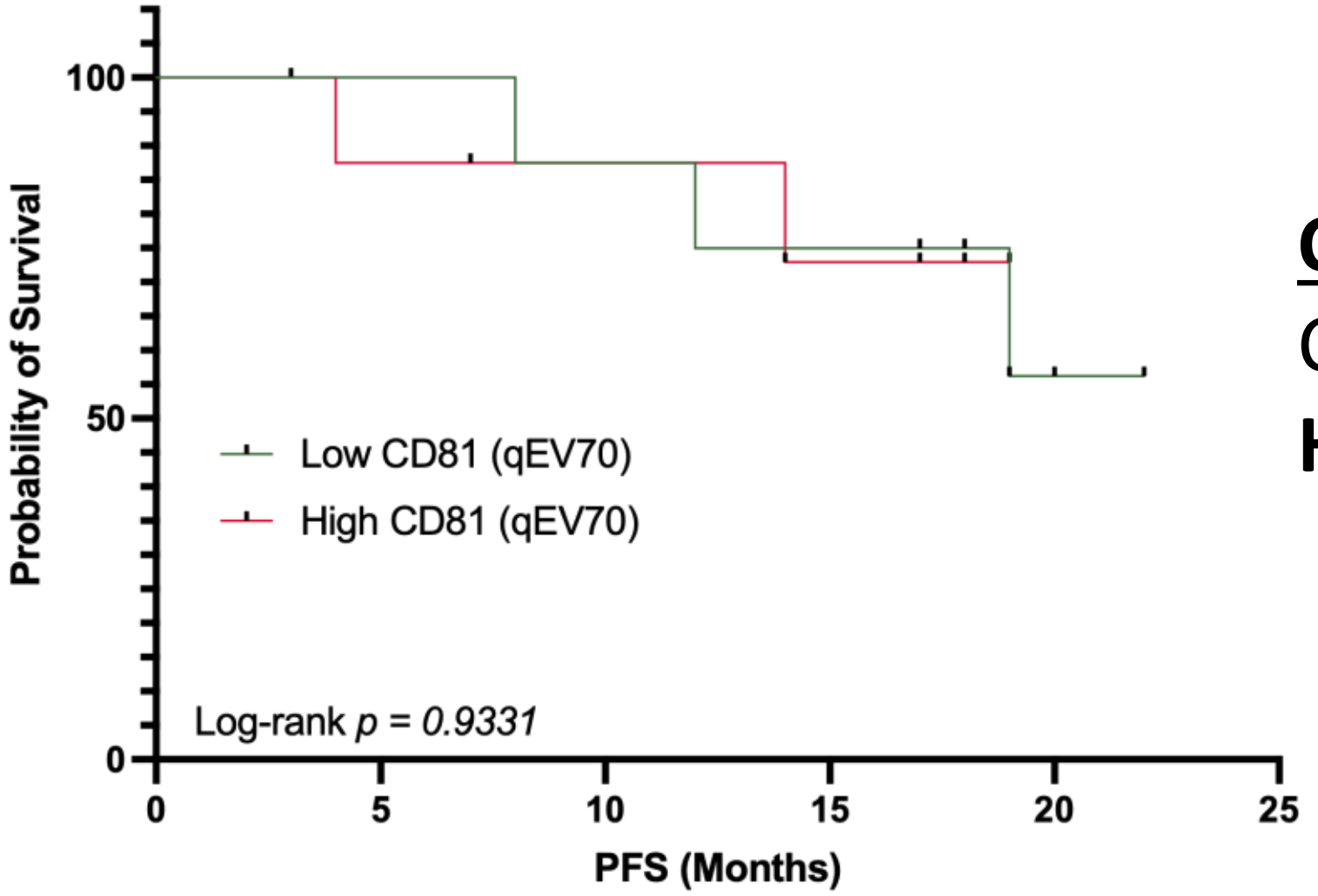

Cox Regression:
CD81, after qEV70:
HR = 0.592

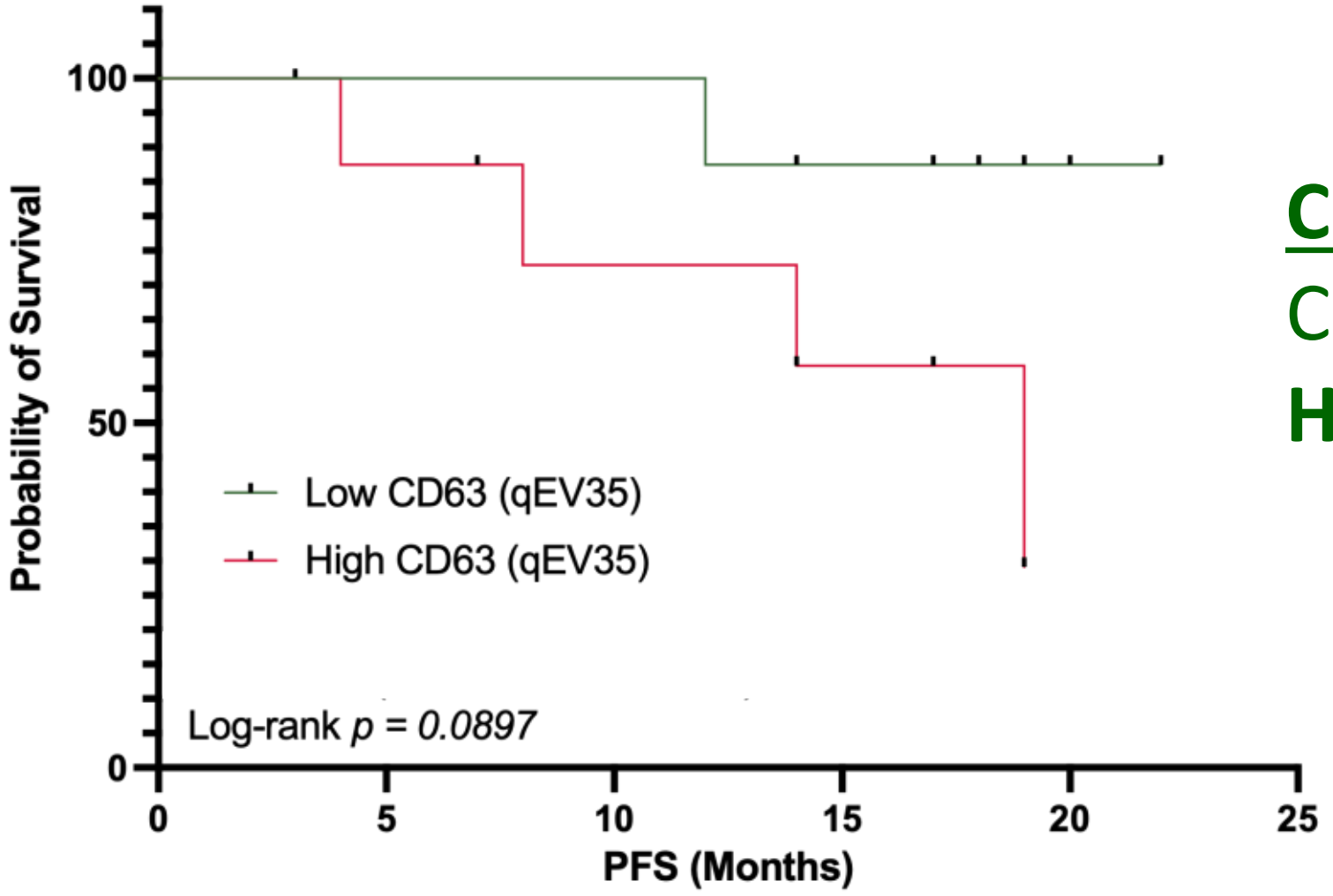

Cox Regression:
CD63, after qEV35:
HR = 5.008

Kaplan Meier: Ovarian cancer

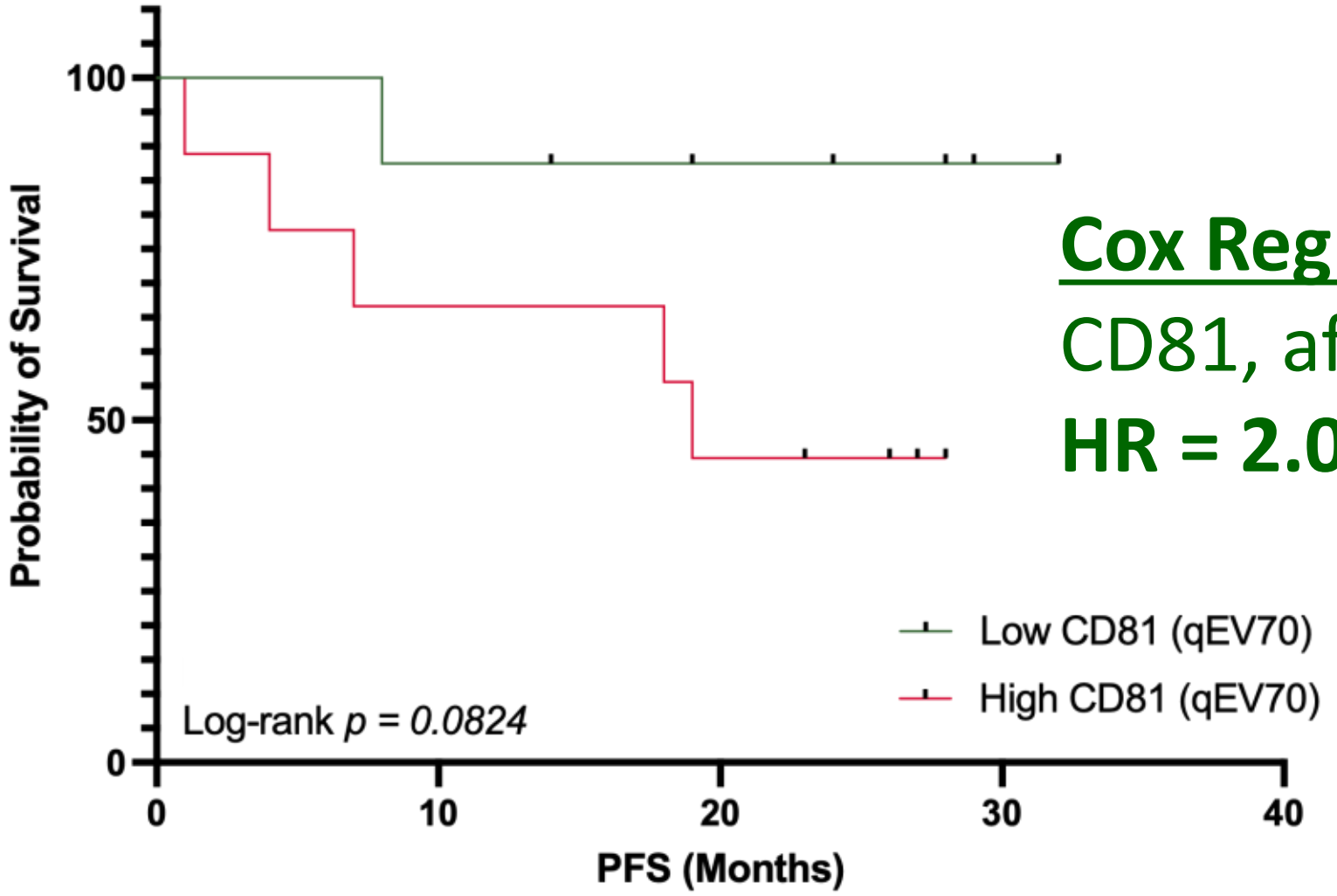

Cox Regression:
CD81, after qEV70:
HR = 2.066

Supplemental Fig. 7. Evaluating potential prognostic value of EVs, by applying Kaplan-Meier curves and Cox regression analyses
